# Biostimulatory Effect of 3-Acetonyl-3-Hydroxyoxindole on the Growth and Secondary Metabolites of *Khaya senegalensis*: UPLC–MS/MS Profiling and Molecular Docking Insights

**DOI:** 10.3390/ijms27157010

**Published:** 2026-08-04

**Authors:** Amr S. Mohamed, Yongdui Chen, Samah M. El-Sayed

**Affiliations:** 1 Botanical Gardens Research Department, Horticulture Research Institute of Agricultural Research Center (ARC), Giza 12619, Egypt; amr1982@arc.sci.eg; 2 Biotechnology and Germplasm Resources Institute, Yunnan Academy of Agricultural Sciences, Kunming 650205, China; 3 Yunnan Provincial Key Laboratory of Agricultural Biotechnology, Kunming 650205, China; 4 Key Lab of South Crop Gene Resources and Germplasm Innovation, Ministry of Agricultural and Rural Affairs, Kunming 650205, China; 5 Ornamental Plants and Woody Trees Department, Agricultural and Biological Research Institute, National Research Centre, Giza 12622, Egypt; ensamah_83@hotmail.com

**Keywords:** *Khaya senegalensis*, 3-acetonyl-3-hydroxyoxindole, UPLC–MS/MS, chalcone synthase, farnesyl diphosphate synthase, molecular docking, bioactive compounds

## Abstract

Developing sustainable agricultural biostimulants that simultaneously optimize vegetative growth and specialized metabolic pathways is critical for maximizing plant growth, photosynthetic efficiency, and metabolome reprogramming. In this study, for the first time, the effects of the biostimulant 3-acetonyl-3-hydroxyoxindole (AHO) on plant growth, photosynthetic efficiency and metabolomics reprogramming were evaluated. The multifaceted effects of AHO (0, 1, 5, 10, and 20 µg/mL) applied via foliar application were evaluated via comprehensive morpho- physiological, UPLC–MS/MS metabolomic and computational docking approaches. AHO positively affects plant growth performance in a concentration-dependent manner. Foliar application at 20 µg/mL produced the maximum vegetative vigor and biomass accumulation, as well as the highest levels of chlorophyll a, chlorophyll b, carotenoids, total flavonoids, and indole contents, while the maximum value of total phenolics was 1 µg/mL. Substantial metabolic flux modulation was confirmed by UPLC–MS/MS profiling, which revealed that 10 µg/mL selectively accumulated chlorogenic acid and rutin, whereas 5 µg/mL preferentially enriched quercetin, quercitrin, limonin and catechin. These empirical metabolic responses are supported by computational docking models, which predict favorable structural interactions between AHO and key biosynthetic enzymes. Insights gained from these integrated morphological, physiological, metabolomic, and computational analyses indicate that AHO applications can effectively improve plant growth and biomass, increase the concentration of bioactive compounds, and enhance the accumulation of bioactive secondary metabolites within the plant.

## 1. Introduction

3-Acetonyl-3-hydroxyoxindole (AHO), an indole derivative isolated from *Strobilanthes cusia*, is a natural plant stimulator that induces systemic acquired resistance (SAR) via salicylic acid (SA)-dependent signaling pathways. Previous studies have indicated its antiviral activity and ability to enhance defense against plant pathogens [1,2]. However, its effects on plant growth under natural environmental conditions, particularly in woody plants, remain unclear.

*Khaya senegalensis* (Desr.) A. Juss., commonly referred to as African mahogany, is a highly valuable multipurpose tree species belonging to the family *Meliaceae* [3]. *K. senegalensis* is a medium-to-large evergreen to semideciduous tree that typically reaches heights of 20–35 m, with a straight cylindrical bole and a dense, rounded crown. The bark is dark gray and fissured, whereas the leaves are pinnately compound, consisting of several pairs of glossy, elliptic leaflets. The species produces small, fragrant, whitish flowers arranged in axillary panicles, followed by woody capsules that release winged seeds adapted for wind dispersal [4]. Due to its distinctive features, it is widely used as an ornamental shade tree in urban landscapes [5]. In addition, it is a fast-growing, drought-tolerant hardwood species indigenous to the savannah and riverine ecosystems of West and Central Africa [6]. Owing to its adaptability to semiarid environments and its high-quality reddish-brown timber, the species has been extensively cultivated in timber plantations. Because of its economic and ecological importance, increasing demand for valuable wood has resulted in overexploitation, leading to population declines and its classification as vulnerable or threatened in parts of its natural distribution range [7]. Phytochemically, *K. senegalensis* is characterized by a rich diversity of secondary metabolites, particularly limonoids (tetranortriterpenoids), which are considered chemotaxonomic markers of the Meliaceae family. The reported compounds include khayanolides, gedunin-type derivatives, flavonoids, tannins, saponins, and other phenolic constituents. These metabolites are associated with a wide range of biological activities, including antimalarial, antimicrobial, anti-inflammatory, antioxidant, and insecticidal effects [8,9,10,11,12].

Chalcone synthase (CHS) and hydroxycinnamoyl-CoA transferase (HCT) regulate critical steps in flavonoid and phenylpropanoid biosynthesis [13], whereas the farnesyl diphosphate synthase FPPS acts as a metabolic gatekeeper for terpenoid accumulation [14]. Additionally, catechol oxidase modulates plant immunity via polyphenolic oxidation [15], collectively serving as pivotal receptors for evaluating AHO-induced metabolic alterations.

In particular, several phenolic and flavonoid compounds, including quercetin, rutin, limonin, chlorogenic acid, and catechin, have been identified or reported in *K. senegalensis*. These compounds play significant physiological and pharmacological roles. For instance, quercetin and rutin are well-known antioxidants that scavenge reactive oxygen species and contribute to stress tolerance and anti-inflammatory activity [16]. Quercitrin, a glycosylated derivative of quercetin, has antimicrobial and cytoprotective effects [17]. Chlorogenic acid functions as a potent antioxidant and is involved in plant defense responses against pathogens and environmental stress [18]. Catechin contributes to antioxidant capacity and exhibits antimicrobial and metal-chelating properties [19]. Moreover, limonin, a representative limonoid, is associated with insecticidal, antifeedant, and anticancer activities [20].

Ultra-performance liquid chromatography–tandem mass spectrometry (UPLC-MS/MS) combines rapid, high-resolution chromatographic separation with sensitive, selective mass spectrometry for the analysis of complex biological samples. In multiple reaction monitoring (MRM) mode, this technique enables the precise identification and quantification of minute quantities of metabolites while minimizing background interference and accelerating analysis compared with conventional methods [21,22,23].

While natural and synthetic indole-derived compounds, such as indole-3-acetic acid (IAA) and indole-3-butyric acid (IBA), are well-established plant growth regulators, their application is often limited by rapid metabolic degradation and broad-spectrum, non-specific signaling. Recent structural activity relationship (SAR) studies suggest that modifying the functional groups on the indole ring can shift the molecule’s role from a standard growth regulator to a targeted stress elicitor. However, the precise biological activity of oxindole derivatives, particularly those with complex C-3 substitutions like 3-acetonyl-3-hydroxyoxindole, remains underexplored. This study addresses this gap by demonstrating how this specific structural modification uniquely triggers secondary metabolite biosynthesis pathways beyond the capacity of traditional indole elicitors.

To date, while the extant literature has predominantly characterized the biological utility of AHO through its role in conferring phytopathogenic disease resistance, the broader physiological capacity of AHO for vegetative performance and specialized metabolism remains largely unexplored. To address this critical knowledge gap, the present study comprehensively evaluated the exogenous effects of various AHO concentrations on the morpho-physiological and secondary metabolic reprogramming of *K. senegalensis* seedlings. By integrating holistic morphological assessments such as plant height, number of leaves, and comprehensive biomass accumulation dynamics with precise physiological monitoring of photosynthetic pigments and nonenzymatic antioxidants, maps of the phenotypic and biochemical shifts induced by AHO can be generated. Furthermore, this empirical framework is supplemented by in silico molecular simulation techniques designed to characterize the specific binding affinities and structural interactions between AHO and key regulatory plant proteins, thereby establishing a predictive structural baseline for understanding potential target interactions and biosynthetic flux modulation of major downstream specialized metabolites.

## 2. Results

### 2.1. Morphological Traits

The data in Table 1 and Table 2 show that some characteristics of *K. senegalensis* seedlings were significantly affected by treatment with different concentrations (0, 1, 5, 10 and 20 µg/mL) of AHO. The highest values were obtained for plant height (38.50 ± 1.67 cm), stem diameter (10.11 ± 0.40 mm), root length (39.50 ± 2.15 cm), fresh mass of leaves, stems and roots (10.93 ± 0.95, 8.86 ± 0.47, and 10.67 ± 0.62 g), respectively, and dry mass of leaves, stems, and roots (5.48 ± 0.24, 3.80 ± 0.31, and 5.10 ± 0.23 g), respectively, in the seedlings exposed to AHO at 20 µg/mL, which was the highest substantial effect compared with the control. The number of leaves was not significantly affected by any of the treatments mentioned.

### 2.2. Biochemical Estimates

**Figure 1:**
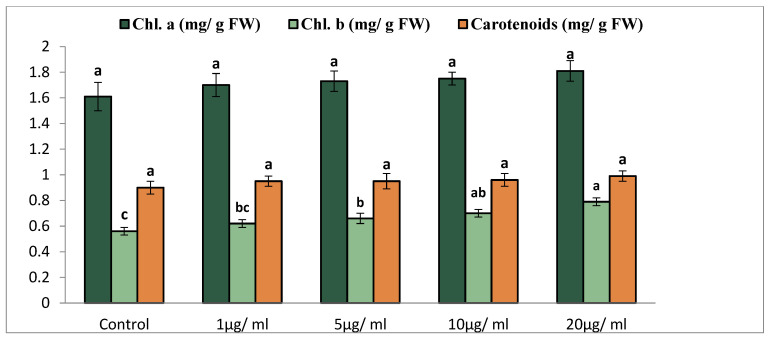
Effects of AHO application on the contents of photosynthetic pigments (chlorophyll a, b and carotenoids) in *K. senegalensis* seedlings. Different lowercase letters in figure indicated significant difference (*p* < 0.05).

The data presented in Figure 1 revealed that the contents of chlorophyll a and carotenoids were not significantly affected by the AHO treatments (*p* = 0.5765 and *p* = 0.6941, respectively) compared with those in the control. In contrast, the chlorophyll b content differed significantly (*p* = 0.0038), demonstrating a distinct response to the treatments. Furthermore, all of these compounds presented their highest contents (chlorophyll a, 1.81 ± 0.08; chlorophyll b, 0.79 ± 0.03; and carotenoids, 0.99 ± 0.04 mg/g FW) at an AHO concentration of 20 µg/mL.

**Figure 2:**
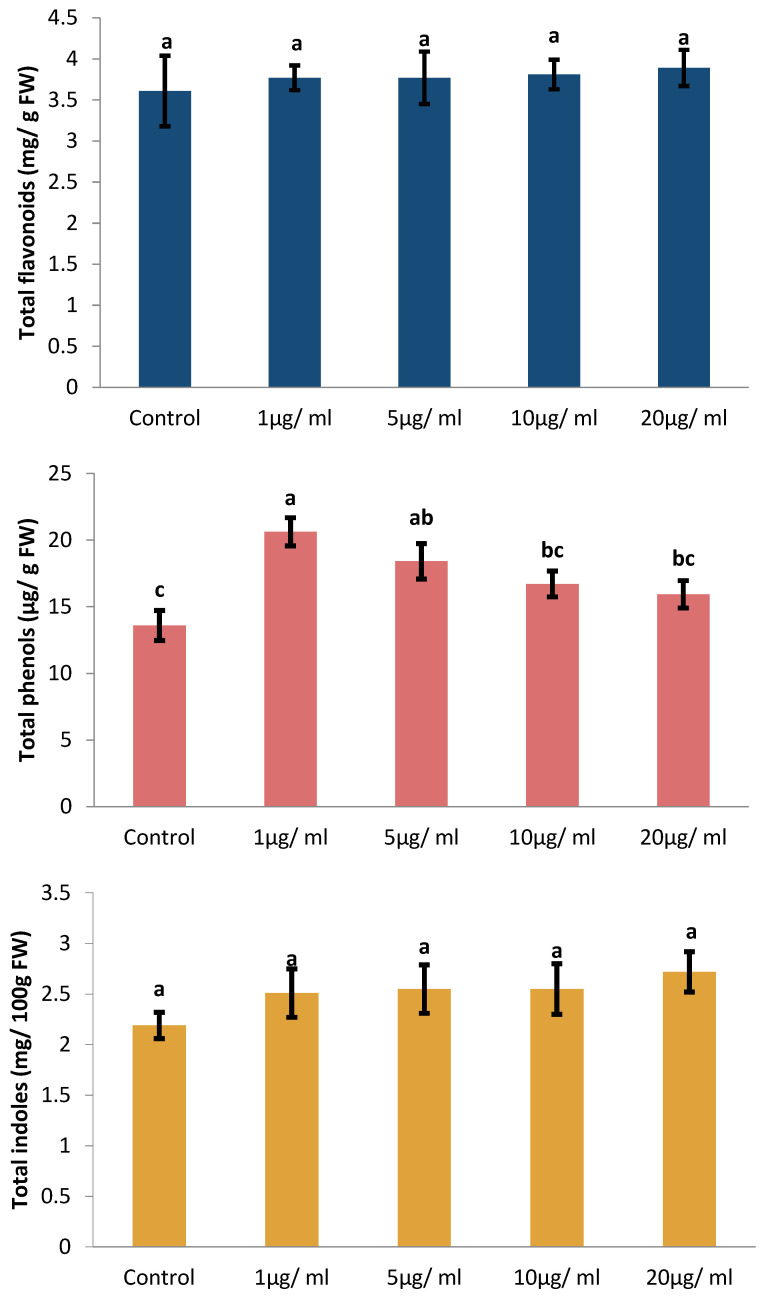
Effects of AHO application on the total flavonoids, total phenols and total indoles contents of *K. senegalensis* seedlings. Note: The lowercase letters in the figure indicate the level of statistical significance compared with the control group.

The data in Figure 2 show that the total contents of flavonoids and indoles were not significantly affected by any of the AHO concentrations (*p* = 0.9650 and *p* = 0.5571, respectively); however, the highest concentrations of flavonoids (3.89 ± 0.22 mg/g FW) and indoles (2.72 ± 0.20 mg/100 g FW) were obtained from seedlings treated with AHO at 20 µg/mL. In contrast, the total phenolic content was significantly affected by AHO (*p* = 0.0119), showing a maximum value of 15.93 ± 1.03 µg/g FW at the 1 µg/mL concentration.

### 2.3. UPLC–MS/MS Profiling of the Detected Phytochemical Compounds

**Figure 3:**
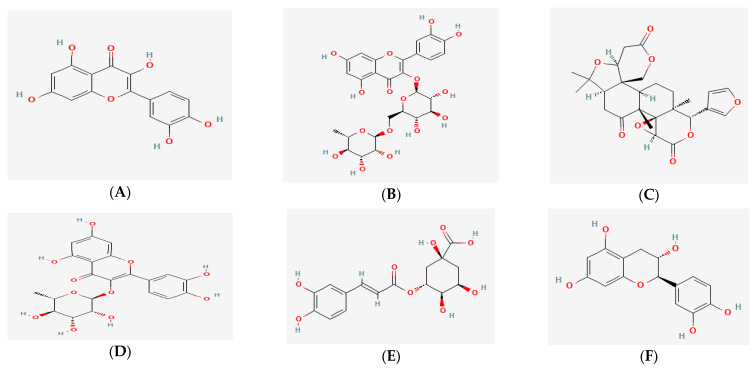
Compound structures of (**A**) quercetin, (**B**) rutin, (**C**) limonin, (**D**) quercitrin, (**E**) chlorogenic acid, and (**F**) catechin in the PubChem database.

Qualitative UPLC–MS/MS data for *K. senegalensis* leaf extracts revealed that six secondary metabolites were highly abundant, with the major compounds being rutin, quercitrin, quercetin, chlorogenic acid, limonin, and catechin Figure 3. These compounds were identified on the basis of their structure, retention time (RT), molecular ion (*m*/*z*) in positive or negative electrospray mode, and molecular formula; Table 3 Characteristic ESI-MS/MS product ion mass spectra of the isolated compounds under positive and negative ionization conditions (Supplementary Figure S1).

Qualitative UPLC–MS/MS analysis enabled the tentative identification of quercetin as a major flavonol in the analyzed sample. Quercetin was detected at a retention time (RT) of 2.48 min, with a molecular ion peak at *m*/*z* 301.0 in negative ionization mode corresponding to the deprotonated ion [M − H]^−^. The observed mass spectral characteristics were consistent with the molecular formula C_15_H_10_O_7_.

As a Flavonoid Glycoside class in the analyzed sample, rutin was detected at a retention time (RT) of 2.13 min, with a molecular ion peak at *m*/*z* 609.5 in negative ionization mode corresponding to the deprotonated ion [M − H]^−^. The observed mass spectral characteristics were consistent with the molecular formula C_27_H_30_O_16_.

Limonin is a derivative of a limonoid class in the analyzed sample. Limonin eluted at a retention time (RT) of 2.79 min, with a molecular ion peak at *m*/*z* 469.2 in negative ionization mode corresponding to the deprotonated ion [M − H]^−^. The observed mass spectral characteristics were consistent with the molecular formula C_26_H_30_O_8_.

Quercitrin is a Flavonoid Glycoside class in the analyzed sample. Quercitrin was detected at a retention time (RT) of 2.22 min, with a molecular ion peak at *m*/*z* 447.1 in negative ionization mode corresponding to the deprotonated ion [M − H]^−^. The observed mass spectral characteristics were consistent with the molecular formula C_21_H_20_O_11_.

Chlorogenic acid is a phenolic acid class, and chlorogenic acid was detected at a retention time (RT) of 2.01 min, with a molecular ion peak at *m*/*z* 353.1 in negative ionization mode corresponding to the deprotonated ion [M − H]^−^. The observed mass spectral characteristics were consistent with the molecular formula C_16_H_18_O_9_.

Catechin is a flavan-3-ol class in the analyzed sample. Catechin was detected at a retention time (RT) of 3.41 min, with a molecular ion peak at *m*/*z* 291.1 in negative ionization mode corresponding to the deprotonated ion [M + H]^+^. The observed mass spectral characteristics were consistent with the molecular formula C_15_H_14_O_6_, confirming the presence of catechin in the extract.

**Figure 4:**
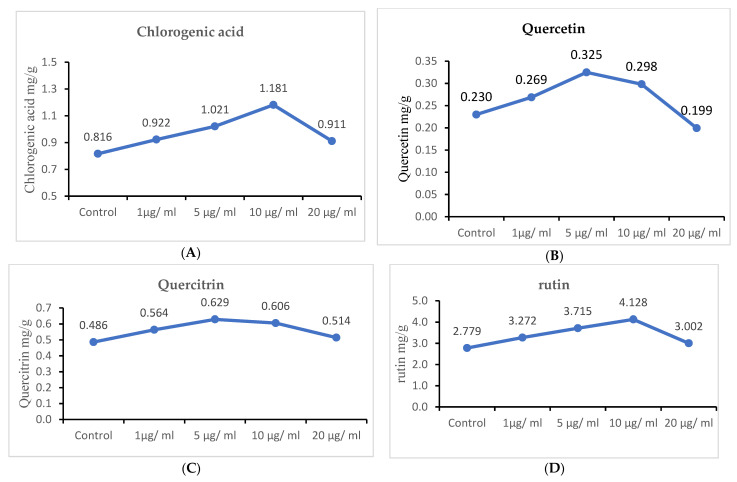

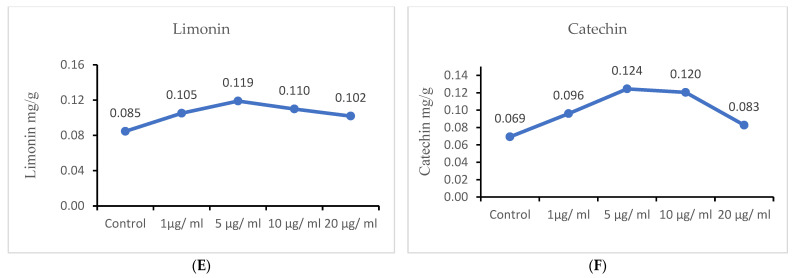
Relative accumulation of (**A**) chlorogenic acid, (**B**) quercetin, (**C**) quercitrin, (**D**) rutin, (**E**) limonin and (**F**) catechin in *K. senegalensis* leaf extracts subjected to different concentrations of AHO.

The data presented in Figure 4A–F were obtained via quantitative UPLC–MS/MS analysis, which revealed significant variations in the levels of secondary metabolites (catechin, rutin, chlorogenic acid, quercetin, limonin, and quercitrin) in the leaf of *K. senegalensis* following treatment with different concentrations of AHO relative to those in the control. The accumulation of these bioactive compounds exhibited a concentration-dependent response to AHO application. Compared with the control treatment, the application of AHO at 10 µg/mL significantly increased the accumulation of chlorogenic acid (1.4-fold) and rutin (1.5-fold).

In contrast, treatment with AHO at 5 µg/mL preferentially increased the levels of quercetin (1.4-fold), quercitrin (1.3-fold), limonin (1.4-fold), and catechin (1.8-fold). These findings indicate that AHO stimulates secondary metabolite biosynthesis in a concentration-dependent manner. However, a subsequent decline in the concentrations of all the detected compounds was observed at the higher concentration of 20 µg/mL.

### 2.4. Molecular Docking of AHO Against Secondary Metabolite Target Enzymes

**Figure 5:**
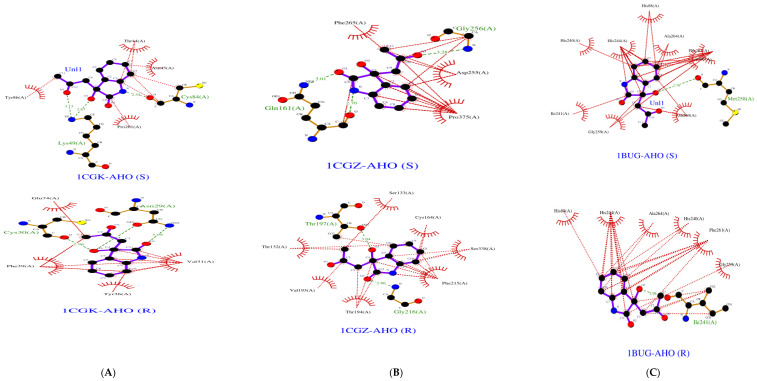

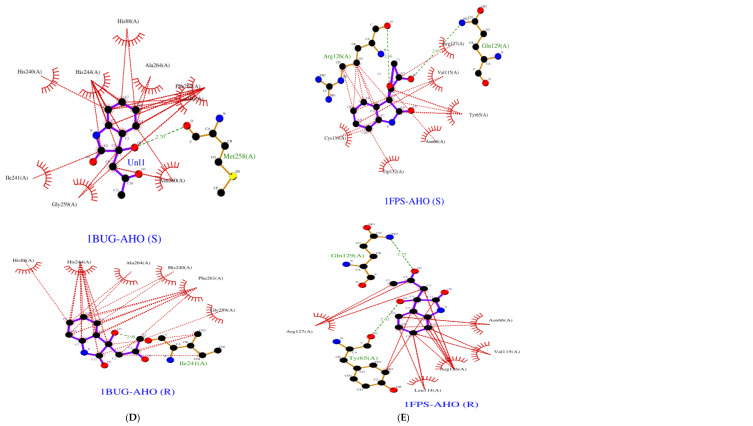
Comparative two-dimensional LigPlot visualizations illustrating the hydrogen-bonding networks and hydrophobic interactions between the *S- and R-enantiomers* configurations of the AHO ligand and key target proteins: (**A**) 1CGK, (**B**) 1CGZ, (**C**) 1BUG, (**D**) 4G0B, and (**E**) 1FPS. Illustrating hydrogen bonds as green dashed lines with distances in Ångstroms and hydrophobic interactions as red dashed lines and arcs between the central ligand and the active site residues.

**Figure 6:**
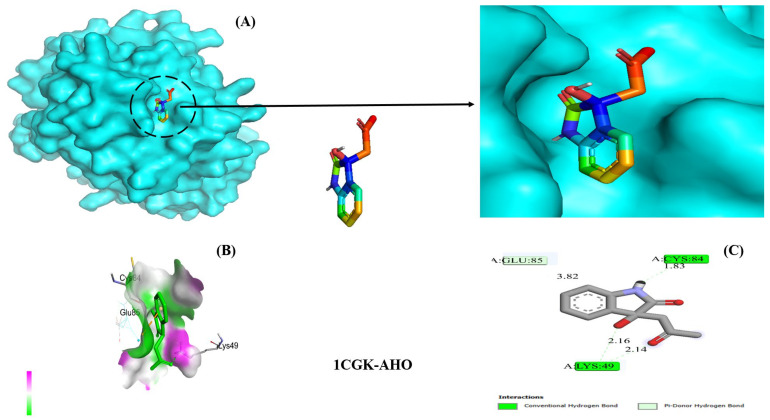

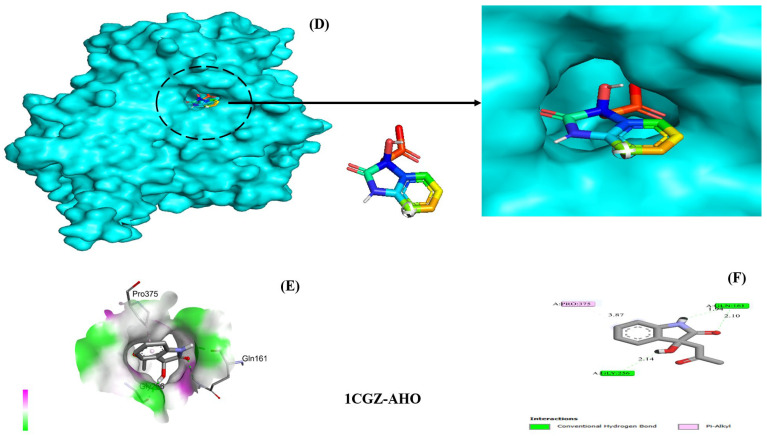

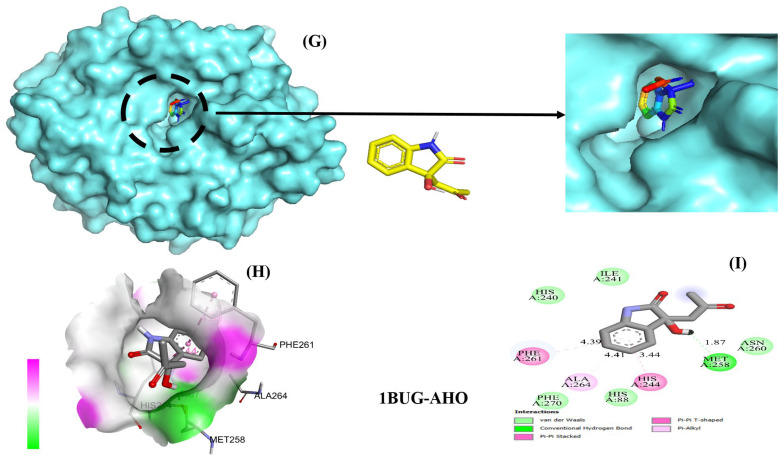

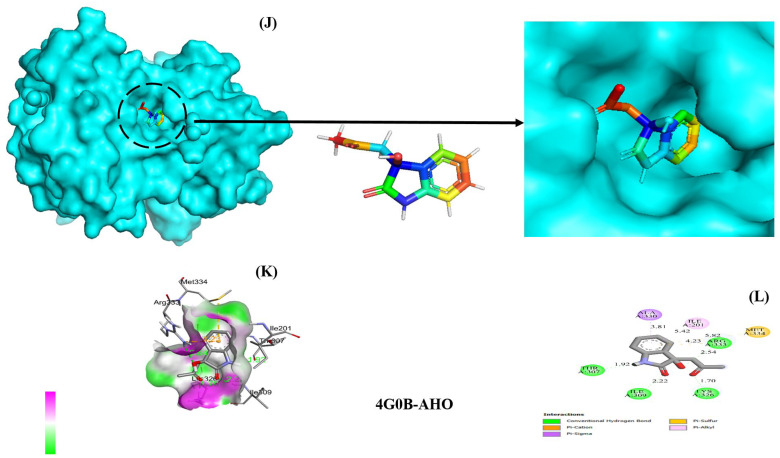

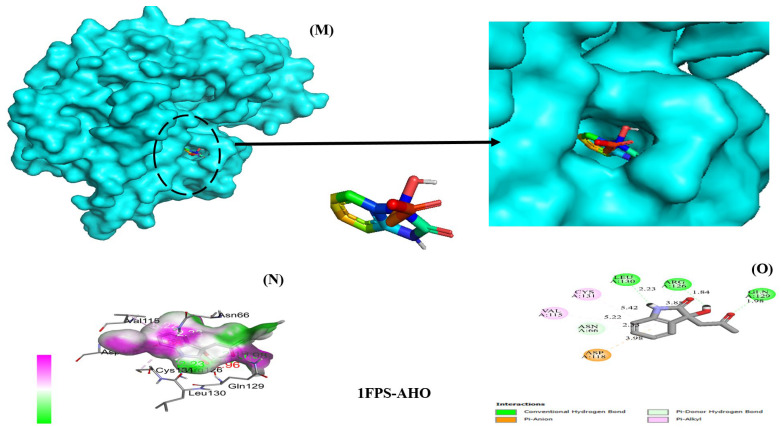
Binding interaction of AHO-(S enantiomer) with multiple receptors (1CGK, 1CGZ, 1BUG, 4G0B, and 1FPS). The subfigures illustrate the 3D binding interactions (**A**,**D**,**G**,**J**,**M**), hydrogen bond surfaces (**B**,**E**,**H**,**K**,**N**), and 2D amino acid interaction profiling (**C**,**F**,**I**,**L**,**O**) of the ligand AHO for each respective receptor.

In silico molecular docking analysis revealed variable binding affinities for the AHO ligand across the evaluated secondary metabolite target enzymes, with calculated binding energies ranging from −7.10 to −5.21 kcal/mol (Table 4). LIGPLOT architectural analysis and protein–ligand interaction profiling (PLIP) verified the successful docking of both the Type S and Type R enantiomers into their respective receptor active sites (Figure 5). Across all primary targets, the Type S configuration consistently demonstrated superior binding capacity relative to its Type R counterpart (Figure 6).

The most robust interaction was recorded against farnesyl diphosphate synthase (FPPS; PDB 1FPS). The 1FPS Type S configuration exhibited the lowest overall binding energy of −7.10 kcal/mol, which corresponded to a predicted inhibition constant (Ki) of 6.25 µM. This complex was structurally stabilized by two hydrogen bonds at Arg126(A) and Gln129(A), alongside hydrophobic contacts involving Tyr65(A), Asn66(A), Val115(A), Cys131(A), and Trp132(A). Conversely, the 1FPS Type R configuration displayed reduced affinity (−6.26 kcal/mol; Ki 25.62 µM) and was maintained by a single hydrogen bond at Gln129(A) coupled with hydrophobic interactions at Tyr65(A), Asn66(A), Leu114(A), Val115(A), Arg126(A), and Arg127(A).

Substantial stabilization was similarly documented within the active site of hydroxycinnamoyl-CoA transferase (HCT; PDB 4G0B). The 4G0B Type S docking pose yielded a binding energy of −6.47 kcal/mol (Ki 18.13 µM) and formed the highest number of hydrogen bonds (four) among all evaluated targets. These hydrogen bonds were located at residues Thr307 (A), Ile309(A), Lys326(A), and Arg333(A), supplemented by hydrophobic and other interactions at Ile201(A) and Ala330(A). The Type R enantiomer presented a weaker interaction profile (−5.82 kcal/mol; Ki 54.59 µM), characterized by a single hydrogen bond at Leu355(A) alongside molecular contacts at Tyr203 and Phe357.

Evaluation of the primary chalcone synthase (CHS) target (PDB 1CGK) indicated that the Type S configuration produced a binding energy of −6.43 kcal/mol (Ki 19.26 µM), mediated by two hydrogen bonds at Lys49(A) and Cys84(A) as well as hydrophobic contacts across four residues (Thr44(A), Asn45(A), Tyr86(A), and Pro201(A)). The 1CGK Type R structure exhibited diminished binding capacity (−5.65 kcal/mol; Ki 71.92 µM), relying on hydrogen bonds at Asn29 (A) and Cys30 (A) with hydrophobic interactions at Val31(A), Tyr36(A), Phe39(A), and Glu74(A).

For catechol oxidase (PDB 1BUG), the Type S ligand achieved a binding energy of −6.35 kcal/mol (Ki 22.15 µM). This interaction was primarily stabilized by one hydrogen bond at Met258 (A) and an extensive hydrophobic network involving His88 (A), His240(A), Ile241(A), His244(A), Asn260(A), Gly259(A), Phe261(A), and Ala264(A). The corresponding 1BUG Type R configuration recorded a lower affinity (−5.89 kcal/mol; Ki 48.46 µM), supported by a single hydrogen bond at Ile241(A) and hydrophobic contacts at His88(A), His240(A), His244(A), Gly259(A), Phe261(A), and Ala264(A).

Finally, docking analysis of the secondary CHS target (PDB 1CGZ) resulted in a binding energy of −5.90 kcal/mol (Ki 47.13 µM) for the Type S configuration, which utilized two hydrogen bonds at Gln161(A) and Gly256(A) combined with hydrophobic interactions at Asp255(A), Phe265(A), and Pro375(A). The 1CGZ Type R configuration demonstrated the lowest overall binding affinity in the dataset at −5.21 kcal/mol, paired with a Ki of 28.14 µM. This specific complex was maintained by two hydrogen bonds at Thr197(A) and Gly216(A), as well as hydrophobic interactions at Thr132(A), Ser133(A), Cys164(A), Val193(A), Thr194(A), Phe215(A), and Ser338(A).

### 2.5. Varimax-Rotated Principal Component Analysis

**Figure 7:**
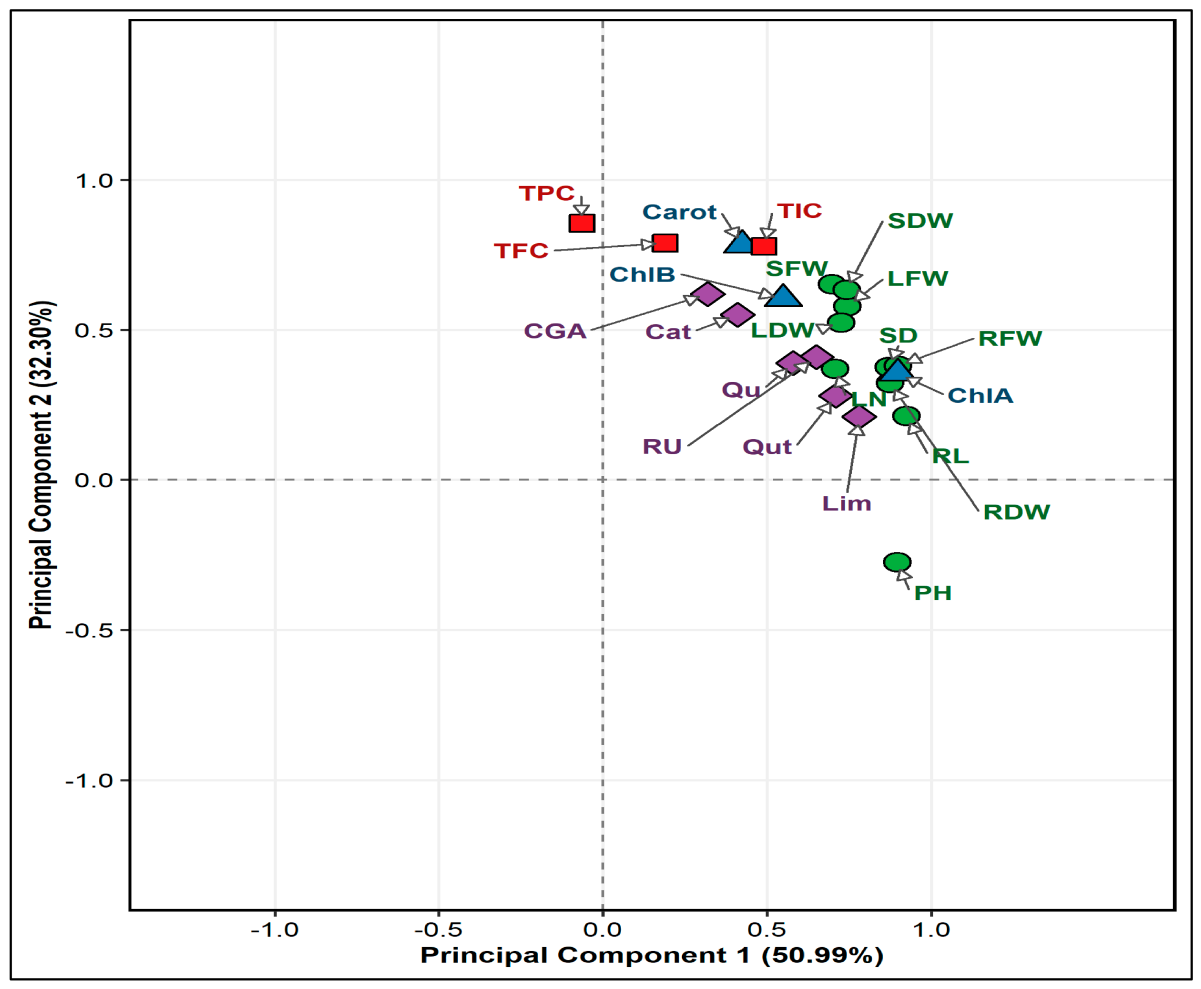
Principal component analysis (PCA) biplot illustrating the relationships among the twenty-two evaluated plant traits. Principal component analysis (PCA) biplot illustrating the relationships among the twenty-two evaluated plant traits. Traits are categorized by color and shape: red squares represent (total phenolic content, total flavonoid content; and total Indole content), blue triangles represent (Chl A; Chl B; and Carotenoids), and green circles indicate (vegetative traits); and squares purples (secondary metabolites).

To evaluate the coordinated shifts and multidimensional relationships among the evaluated traits, a varimax-rotated principal component analysis (PCA) (Figure 7) was conducted, which captured 83.29% of the total dataset variance. The twenty-two variables traits were successfully segregated into distinct, highly interpretable functional modules within a two-dimensional spatial configuration. Principal component 1 (PC1) accounted for the majority of the total variance (50.99%) and was heavily dominated by primary structural traits, displaying strong positive orthogonal loadings for root development parameters [root length (RL) and root dry weight (RDW)], root fresh weight (RFW), and chlorophyll a (ChlA). This alignment established PC1 as a primary indicator of vegetative vigor and structural biomass accumulation. Concurrently, Principal Component 2 (PC2) captured 32.30% of the remaining variance and served as a representative axis for biochemical quality and adaptive antioxidant defense systems. High positive loading coordinates on PC2 were explicitly driven by secondary metabolism, where global antioxidant indices [total phenolic content (TPC), total flavonoid content (TFC), carotenoids (Carot), and Total Indole Content (TIC)] clustered tightly with individual secondary metabolites [chlorogenic acid (CGA), catechin (Cat), rutin (RU), quercetin (Qu), and quercitrin (Qut)], strongly suggesting a highly coordinated accumulation of metabolites within the phenylpropanoid pathway. Crucially, the spatial distribution of variables within the rotated vector space revealed critical biological trade-offs and resource partitioning dynamics. Plant height (PH) exhibited unique spatial decoupling by localizing to the lower-right quadrant; while it maintained a robust positive loading on PC1, it displayed a nearly orthogonal (90-degree) geometric orientation relative to the primary phenolic clusters (TPC and TFC) on the upper horizon. This perpendicular orientation statistically confirmed a complete lack of correlation, demonstrating that vertical stem elongation and protective polyphenol synthesis operate via independent metabolic pathways. Conversely, intermediate bridging coordinates between the structural vectors of PC1 and the chemical vectors of PC2 were occupied by chlorophyll b (ChlB), stem fresh weight (SFW), and leaf dry weight (LDW). This intermediate allocation structurally reflects the biological dependence of secondary metabolite synthesis on primary carbon skeletons generated through photosynthetic resource partitioning, confirming that the plant successfully channels primary fixed carbon into protective phenylpropanoid pathways without inducing vegetative retrogression.

## 3. Discussion

The present study provides a comprehensive evaluation of the indole derived biostimulant AHO and its multifaceted effects on plant growth, biochemical constituent performance, and secondary metabolic flux. The integration of vegetative growth parameters, biochemical profiling, and the accumulation of secondary metabolites, along with in silico molecular docking analysis, offers a predictive structural and physiological framework for understanding how the AHO compound can influence plant systems. Overall, these findings support the hypothesis that this indole derivative functions as a biostimulant with potential hormone-like activity and a capacity to enhance secondary metabolism.

Importantly, to the best of our knowledge, no previous studies have investigated the effects of this AHO on plant growth mechanisms. Therefore, we attempted to highlight the novelty and significance of AHO and its role in vegetative growth, photosynthesis, phenols, flavonoids, and indoles, in addition to other bioactive compounds in *Khaya senegalensis*, as an initial contribution toward understanding this new class of bioactive compounds.

### 3.1. Effects of AHO on the Growth of K. senegalensis Seedlings

According to our results in Table 1 and Table 2, seedlings exposed to AHO at 20 µg/mL presented the highest values for all traits, including plant height, stem diameter, number of leaves, root length, fresh mass (leaves, shoots and roots), and dry mass (leaves, shoots and roots), compared with those of untreated seedlings. The observed increases in plant height, stem diameter, root elongation and biomass accumulation suggest that AHO may act as an auxin mimic or modulator of auxin signaling pathways, meaning that it triggers systemic physiological responses [24].

Previous studies have reported the presence of indole containing metabolites, which may indicate the activation of tryptophan-dependent pathways associated with indole-3-acetic acid (IAA) biosynthesis and signaling, which are closely linked to cell elongation, cell division, vascular differentiation, and biomass accumulation in plants [25,26,27,28].

### 3.2. Biochemical Estimates

#### 3.2.1. Photosynthetic Pigments

The results revealed that the application of AHO promoted the accumulation of photosynthetic pigments, including chlorophyll a, chlorophyll b, and carotenoids, relative to the untreated control. While chlorophyll a and carotenoid contents did not differ significantly across AHO concentrations, chlorophyll b content increased markedly at 20 µg/mL, suggesting that indole exerts a concentration-dependent influence on chlorophyll b biosynthesis. The overall increase in pigment levels may reflect improved stability of the photosynthetic machinery and activation of pigment biosynthetic pathways. Indole is integral to the tryptophan-dependent biosynthetic pathway and serves as the primary precursor of indole-3-acetic acid (IAA). Elevated IAA signaling upregulates the expression of key genes that govern chloroplast development, photosynthetic efficiency, and chlorophyll biosynthesis. Specifically, this cascade modulates downstream molecular mechanisms, including the activation of chlorophyll synthase and the upregulation of genes within the tetrapyrrole pathway, which directly dictates chlorophyll production [29,30,31]. Wei et al. [32] reported that 3-hydroxy-2-oxindole, an indole derivative with a disulfide structure, can promote photosynthesis by increasing the chlorophyll content. In addition, indole-related signaling may increase the expression of light-harvesting complex (LHC) proteins, which are essential for stabilizing chlorophyll molecules, particularly chlorophyll b, and improving energy capture efficiency. The observed differences in chlorophyll content among the treatments, particularly chlorophyll b, may be attributed to the functional role of chlorophyll b in light absorption and energy transfer. This, in turn, is fundamentally associated with enhanced photosynthetic efficiency and potential adaptation to fluctuating light conditions, ultimately contributing to overall photosynthetic performance through the modulation of chloroplast metabolic activity [33].

#### 3.2.2. Nonenzymatic Antioxidants

The findings of the present study demonstrated that the flavonoid and indole contents increased with increasing concentrations of AHO, whereas seedlings presented high flavonoid and indole contents at AHO concentrations of 20 µg/mL. Compared with that of the control, the total phenol content of *K. senegalensis* increased in response to all the AHO concentration application treatments, which is in agreement with the findings of Wei et al. [32], who reported that indole scaffolds are structurally and functionally relevant in plant biology because of their close association with endogenous auxins.

Unexpectedly, we observed that AHO at 1 µg/mL had the most significant effect and resulted in the highest phenol content compared with the other concentrations of AHO, which may be related to the fact that AHO can activate the PAL metabolic pathway and thereby produce more phenolic compounds [1]. Refs. [34,35] stated that indole derived compounds may enhance PAL gene expression through reactive oxygen species (ROS)-mediated signaling and hormonal crosstalk involving salicylic acid (SA), jasmonic acid (JA), and auxin-related pathways [36,37]. Mechanistically, this response may be associated with improved antioxidant capacity, which subsequently promotes PAL activity and subsequently promotes metabolic flux through the phenylpropanoid pathway, leading to increased biosynthesis of hydroxycinnamic acids, flavonoids, and other antioxidant phenolic metabolites associated with plant defense and stress adaptation [38].

#### 3.2.3. Secondary Metabolites

Our data show that UPLC/MS-MS analysis revealed differences in metabolite composition and variations in quantities among treatments exposed to different concentrations of AHO. The metabolic response followed a pattern on the basis of concentration. We observed clear changes in metabolite accumulation as AHO concentration increased from low to high. At 5 µg/mL, we observed the highest accumulation levels of quercetin, quercitrin, catechin, and limonin. In contrast, rutin and chlorogenic acid reached their maximum levels at 10 µg/mL; these findings are consistent with previous hypotheses [39,40,41] suggesting that indole derivatives modulate the metabolic flux between the shikimate and phenylpropanoid pathways. The shikimate/phenylpropanoid network represents the common biosynthetic origin for several detected metabolites, including quercetin, quercitrin, rutin, chlorogenic acid, and catechin. As a central metabolic system in plants, these pathways rely on a shared primary carbon pool, making them highly sensitive to regulatory signals and metabolic feedback mechanisms. Indole-derived compounds are therefore proposed to function as metabolic regulators capable of acting as transcriptional modulators, homeostatic signaling molecules, and feedback regulators of secondary metabolism [42]. Conversely, McGarvey and Croteau, 1995 and Leone et al. 2007 [43,44] reported that hormonal signaling mediated by low-dose indole indirectly modulates the cytosolic acetate mevalonate (MVA) pathway, accelerating the metabolic flux from acetyl-CoA to squalene and eventually to limonin biosynthesis.

In particular, elevated intracellular levels of indole derivatives may influence the activity and expression of phenylalanine ammonia lyase (PAL), the key rate-limiting enzyme responsible for converting phenylalanine into cinnamic acid, which represents the entry point for phenylpropanoid biosynthesis. Modulation of PAL activity directly affects downstream phenylpropanoid production and, consequently, alters the accumulation profile of flavonoids and related secondary metabolites [45,46,47].

Increased PAL activity increases metabolic flux toward the synthesis of phenolic acids, flavonoids, tannins, lignin precursors, and anthocyanins [48,49]. Indole-derived compounds may also stimulate downstream enzymes such as chalcone synthase (CHS), chalcone isomerase (CHI), and cinnamate-4-hydroxylase (C4H), thereby promoting flavonoid biosynthesis and accumulation [50,51].

The application of AHO acts as a powerful biochemical trigger that initiates a distinct upstream metabolic flux within the plant. Upon treatment, AHO application is associated with an increased accumulation of downstream phenylpropanoids [2], which is the master gateway enzyme of the phenylpropanoid pathway. This enzymatic modulation may facilitate the conversion of amino acid precursors into an enriched pool of phenylpropanoids, suggesting a partitioning of downstream secondary metabolism into two major defense pathways. On one branch, total phenolic concentrations spike, providing the raw chemical building blocks for physical cell wall fortification via lignification and the synthesis of specialized, localized phytoalexin toxins. Simultaneously, the alternative branch drives a major accumulation of flavonoids, which migrate to stress points to serve as potent reactive oxygen species (ROS) scavengers and to disable vital pathogen enzymes through chemical chelation.

Numerous studies demonstrate that the application of biostimulants such as seaweed extracts, protein hydrolysates, humic substances, beneficial microorganisms, chitosan, and amino acids significantly increases the accumulation of bioactive compounds, including phenolic acids, flavonoids, anthocyanins, terpenoids, and alkaloids across various horticultural and medicinal plants [52,53]. These sustainable agricultural inputs actively enhance the biosynthesis of phenolic acids, thereby improving plant defense mechanisms, antioxidant capacity, and overall nutritional quality. Among the most extensively studied elicitors, seaweed extracts (particularly from Ascophyllum nodosum and Ecklonia maxima) consistently promote the accumulation of chlorogenic, caffeic, ferulic, and p-coumaric acids by stimulating phenylpropanoid metabolism [54]. Similarly, chitosan, a natural polysaccharide, is widely recognized for triggering defense-related signaling pathways and driving the accumulation of caffeic, ferulic, rosmarinic, and chlorogenic acids [55]. Furthermore, recent agricultural trials demonstrate that the application of GABA significantly elevates endogenous pools of total flavonoids and total phenolics in Celosia argentea by 1.6- and 2.8-fold, respectively [56].

### 3.3. Molecular Docking of AHO Against Secondary Metabolite Target Enzymes

In silico molecular docking provides critical structural insights into the biomolecular interactions governing the putative regulatory activity of the AHO ligand. The current architectural mapping indicates a pronounced stereoselective preference, with the Type S configuration consistently exhibiting superior binding capacity across all evaluated secondary metabolite target enzymes compared to its Type R enantiomer. This strong stereospecificity suggests that the precise spatial orientation of the AHO scaffold is a primary determinant for optimal accommodation and electronic stabilization within these highly conserved catalytic cavities.

The most robust intermolecular interaction was observed within the active site of farnesyl diphosphate synthase (FPPS; PDB 1FPS), a pivotal regulatory enzyme in isoprenoid metabolism. The high-affinity binding of the AHO-(S) enantiomer is driven by focused polar anchoring through Arg126 and Gln129, complemented by a distinct hydrophobic envelope encompassing residues such as Tyr65, Val115, and Trp132. This structural coordination is essential for controlling chain length specificity and stabilizing intermediate precursors. This aligns with the established role of FPPS in providing C15 farnesyl precursors for essential plant terpenes and steroids [57,58]. The marked preference for the Type S configuration indicates that specific spatial constraints and hydrophobic boundaries within the FPPS pocket tightly govern ligand entry and stabilization.

Strong structural stabilization was similarly documented for hydroxycinnamoyl-CoA transferase (HCT; PDB 4G0B), a key rate-limiting enzyme in the phenylpropanoid biosynthetic pathway. The docking profiles reveal that the AHO-(S) ligand forms a highly stable complex mediated by the densest hydrogen-bonding network observed in this study (Thr307, Ile309, Lys326, and Arg333). This extensive polar coordination likely serves to anchor the ligand effectively against the polar microenvironment of the enzyme, hypothetically facilitating the metabolic flux required to redirect hydroxycinnamoyl-CoA thioesters toward downstream phenolic and flavonoid biosynthesis.

Within the primary chalcone synthase (CHS) catalytic system (PDB 1CGK) which initiates the critical rate-limiting assembly of the fundamental flavonoid scaffold [59]. AHO-(S) configuration demonstrated highly favorable structural integration. This interaction is mediated by conserved hydrogen bonds at Lys49 and Cys84, reinforced by localized hydrophobic contacts (Thr44, Asn45, Tyr86, Pro201). The empirical interaction profiling suggests that AHO-(S) successfully mimics natural substrates through precise polar and hydrophobic matching. This positions the ligand favorably to influence the proper folding profile and Claisen cyclization of tetraketide intermediates, which may ultimately regulate the formation of naringenin chalcone [60,61].

Complementing this, docking against the secondary CHS target (PDB 1CGZ) further supported the role of polar and hydrophobic synergy in ligand recognition. The AHO-(S) configuration utilized highly directional hydrogen bonds at Gln161 and Gly256 (1.94 Å and 2.14 Å, respectively), paired with a restrictive hydrophobic environment created by residues including Pro375. This structural arrangement promotes efficient ligand desolvation and significantly reduces the energetic penalties associated with binding, thereby enhancing both product specificity and the conformational integrity of the catalytic active site [60,62,63].

For catechol oxidase (PDB 1BUG), a copper-dependent enzyme responsible for the catabolic turnover and oxidative browning of phenolic compounds into reactive quinones [64,65,66], the AHO-(S) ligand achieved stable anchoring via a single hydrogen bond at Met258 alongside a massive, continuous hydrophobic network (His88, His240, Ile241, His244, Asn260, Gly259, Phe261, Ala264). This interaction pattern closely mirrors conserved tyrosinase architectures, where broad hydrophobic clustering promotes ligand desolvation and restricts access to the dicopper catalytic center. The robust hydrophobic confinement observed for AHO-(S) effectively excludes water molecules from the pocket, lowering the localized dielectric constant and optimizing intermolecular stabilization.

Collectively, these structural profiling data suggest that the AHO-(S) ligand adopts a dual-action, highly coordinated binding mode. By effectively binding to and potentially modulating key biosynthetic enzymes (CHS, HCT, FPPS) while simultaneously occupying the active site of oxidative degradation enzymes (catechol oxidase), AHO emerges as a promising chemical candidate for metabolic engineering. This stereoselective targeting approach may serve to stabilize the dynamic balance of plant secondary metabolites, shielding existing antioxidant pools from copper-catalyzed degradation while regulating the upstream flux of flavonoids and isoprenoids.

We further demonstrate that these theoretical docking models are consistent with our experimental UPLC-MS/MS findings. Specifically, the measured increases in targeted secondary metabolites including chlorogenic acid, rutin, quercetin, and limonin offer biochemical support for metabolic pathway shifts that closely correlate with the structural docking predictions for CHS, HCT, FPPS, and catechol oxidase.

Beyond the computational structural scale, the physiological implications of these treatments were evaluated in vivo. The application of varimax-rotated principal component analysis (PCA) successfully decoupled the complex physiological network of the evaluated plant material into clear, functionally independent modules. The analysis demonstrated that vegetative growth traits (PC1) and adaptive secondary metabolic defense systems (PC2) exhibit distinct statistical variance profiles, accounting for 83.29% of the total dataset variance. The close spatial synchronization among global antioxidant indices and individual phenolic fractions suggests that specific flavonoid allocations are primary contributors to the overall antioxidant potential of the plant, reflecting a highly coordinated accumulation of metabolites within the phenylpropanoid pathway [67,68,69].

## 4. Materials and Methods

### 4.1. Plant Material, Location and Experimental Procedures

Seeds of *Khaya senegalensis* were germinated at Yunnan Academy of Agricultural Sciences (Kunming, China) during March 2024 under greenhouse conditions (16 h of light at 30 ± 2 °C and 8 h of darkness at 20 ± 2 °C). The seeds were sown in 30 cm diameter plastic pots (seed/pot), which were filled with a growing medium consisting of peat moss, perlite, and clay in equal volumes. The chemical properties of the growing medium were as follows: the chemical properties of the soil used in the experiment were conducted according to the methods of the Ministry of Agriculture and Rural Affairs of the People’s Republic of China [70,71], and those of the Ministry of Ecology and Environment of the People’s Republic of China (2016) were as follows: pH = 6.31; EC25 = 159 µS/cm; soil organic matter = 77.50 g/kg; Na^+^ = 0.120 cmol/kg; K^+^ = 0.188 cmol/kg; Ca^2+^ = 0.328 cmol/kg; Mg^2+^ = 0.0785 cmol/kg; Cl^−^ = 0.148 cmol/kg; and Co_3_^−^ = 0.0476 cmol/kg.

After seedlings germinated and two true leaves emerged, seedlings were treated with AHO as foliar application at different concentrations (CK: 0 µg/mL (control); T1: 1 µg/mL; T2: 5 µg/mL; T3: 10 µg/mL; and T4: 20 µg/mL). AHO was applied twice during the growing season, the first applied one month after planting, and the second applied one month later. After six months, seedlings were harvested and subjected to the required analyses.

#### Preparation of AHO

One gram of AHO powder was dissolved in 1 mL of dimethyl sulfoxide (DMSO) and then diluted with distilled H_2_O to a final volume of 100 mL as a stock solution. Then, the solution was diluted to the appropriate concentration before use as needed [1].

### 4.2. Vegetative Growth Parameters

The parameters of plant height (cm), stem diameter (mm), number of leaves, root length (cm), and fresh and dry mass of leaves, stems and roots (g) were determined.

### 4.3. Biochemical Estimates

#### 4.3.1. Photosynthetic Pigments (mg. g^−1^ F.W.)

A known weight (0.1 g) of fresh leaves was crushed with 10 mL of 80% acetone (*v*/*v*). The supernatant was then used to measure the contents of chlorophyll a, b, and carotenoids via a spectrophotometer at wavelengths of 663, 647, and 470 nm [72].

#### 4.3.2. The Total Flavonoid Content (mg/g F.W.)

Orsavová et al. [73] employed the aluminum chloride colorimetric method to determine the total flavonoid concentration of the ethanol extract of fresh leaf tissue.

#### 4.3.3. Total Phenol Content (µg/g FW)

Total phenolic content was estimated via the method published by [74]. The ethanol extract of fresh leaves was treated with Folin–Ciocalteu reagent and sodium carbonate (14%). The optical density was measured at a wavelength of 765 nm and calculated using the gallic acid standard curve.

#### 4.3.4. Determination of Total Indoles Content (mg/100 g FW)

Total indoles content was determined according to [75]. Fresh tissue (0.5 g) was extracted with 80% ethanol and centrifuged. An aliquot of the extract was mixed with Salkowski reagent and incubated in the dark for 30 min. Absorbance was measured at 530 nm, and indole content was calculated using an indole-3-acetic acid standard curve. The results are expressed as mg/100 g fresh weight.

### 4.4. Ultra-Performance Liquid Chromatography–Tandem Mass Spectrometry (UPLC-MS/MS)

#### 4.4.1. Chemicals and Reagents

LC–MS grade methanol, acetonitrile, formic acid, and ammonium formate (purity ≥98%) were purchased from Macklin (Shanghai, China). Distilled water (ELGA, High Wycombe, UK) was used throughout the study. All sol-vents were filtered and degassed prior to use.

#### 4.4.2. Sample Preparation

The plant samples were ground into a fine powder under liquid nitrogen and thoroughly homogenized. Approximately 0.05–0.1 g of each sample was transferred into a 15 mL centrifuge tube, followed by the addition of 2 mL of methanol. Extraction was carried out using ultrasonic treatment at 4 °C for 1 h. The extracts were then centrifuged at 10,000 rpm for 10 min, and the resulting supernatants were collected and filtered through a 0.22 µm membrane filter. The filtrates were diluted with distilled water (1:1, *v*/*v*) and vortexed prior to analysis. When necessary, further dilution was performed with 50% methanol to ensure that analyte concentrations fell within the calibration range.

Quality control (QC) samples were prepared following the same procedure as the standards. All sample preparation steps were conducted on wet ice and protected from light to minimize degradation.

Chromatographic separation was performed using a Waters UPLC system (Waters, Milford, MA, USA) equipped with a BEH C18 column (2.1 mm × 100 mm, 1.7 µm). The column temperature was maintained at 35 °C. The mobile phase consisted of water containing 0.1% formic acid (solvent A) and acetonitrile containing 0.1% formic acid (solvent B).

The gradient elution program was as follows: 0–2 min, 5% B; 2–10 min, 5–40% B; 10–15 min, 40–90% B; 15–17 min, 90% B; 17–18 min, 90–5% B; and 18–20 min, 5% B for column re-equilibration. The flow rate was set at 0.3 mL min^−1^, the autosampler temperature was 10 °C, and the injection volume was 10 µL.

Mass spectrometric detection was performed using a Waters TQD LC-MS/MS (Waters, Milford, MA, USA) equipped with an electrospray ionization (ESI) source operating in both positive and negative ion modes. The optimized parameters were as follows: ion spray voltage of 4500 V in positive mode and −4500 V in negative mode, source temperature of 500 °C, curtain gas set at 25 psi, nebulizer gas (GS1) at 50 psi, and auxiliary gas (GS2) at 50 psi.

Quantitative analysis was conducted in multiple reaction monitoring (MRM) mode, with electrospray ionization operated primarily in negative mode (ESI^−^). Compound-specific MRM transitions were optimized for each flavonoid standard. In addition, the collision energy (CE) and fragmentor voltage for each analysis were individually tuned to achieve maximum signal intensity and enhance quantitative accuracy.

All other mass spectrometric conditions were maintained as described above. Data acquisition and processing were performed using MassLynx version V4.1. Calibration curves were constructed using external standards, and the concentrations of flavonoids in the samples were calculated on the basis of peak areas. The resulting data were further analyzed to evaluate the molecular characteristics and relative abundances of the detected compounds. Analytical method validation parameters for the targeted metabolites (Table S1); calibration curves (Figure S2).

### 4.5. Molecular Docking

#### 4.5.1. Ligand Preparation

The three-dimensional (3D) structure of AHO (Conformer3D_COMPOUND_CID_36460) was retrieved from the PubChem Database (Table 5). The AHO utilized as the raw material for all biological assays was applied as a racemic mixture; thus, the observed physiological responses reflect the combined efficacy of both stereoisomers. To rigorously evaluate the stereoselective binding dynamics of this racemate at the molecular level, the 3D coordinates for both the R- and S-enantiomers were explicitly generated from their respective stereospecific SMILES strings CC(=O)C[C@]1(C2=CC=CC=C2NC1=O)O and CC(=O)C[C@@]1(C2=CC=CC=C2NC1=O)O). To prepare the AHO enantiomers as ligands for docking, geometry optimization and energy minimization were performed using the Open Babel software suite version 2.4.1 [76]. The structures were subsequently processed using AutoDock Tools (ADT, Version 1.5.7), where polar hydrogen atoms were added, nonpolar hydrogen atoms were merged, and Kollman charges were assigned. The finalized ligand coordinate file was then exported in the standard PDBQT format.

#### 4.5.2. Protein Preparation

To evaluate the binding affinities and structural interactions of AHO, we targeted four central enzymes within the flavonoid, phenylpropanoid, and terpenoid biosynthetic pathways: chalcone synthase (CHS), catechol oxidase, hydroxycinnamoyl-CoA transferase (HCT), and farnesyl diphosphate synthase (FPPS). Target selection was empirically supported by our UPLC-MS/MS data, which confirmed the active accumulation of rutin, quercetin, catechin, quercitrin, chlorogenic acid, and limonin.

Lacking species-specific crystallographic data for *Khaya senegalensis*, we utilized high-resolution surrogate templates from the Protein Data Bank (PDB). Given the strict evolutionary conservation of these catalytic pockets, structures derived from Medicago sativa, Ipomoea batatas, and Coffea canephora (PDB IDs: 1CGK, 1CGZ, 1BUG, and 4G0B) served as highly reliable structural proxies. For FPPS, the Gallus gallus model (PDB ID: 1FPS) was employed, as the core isoprenoid catalytic architecture is conserved across eukaryotes (Table 6). Receptor preparation was performed using AutoDock Tools (v.1.5.7). Crystallographic water molecules, cofactors, and native ligands were systematically removed from each template. Polar hydrogen atoms and Kollman charges were subsequently added to optimize electrostatic properties, and the final structures were exported in PDBQT format for docking simulations.

### 4.6. Statistical Analysis

The experiment was laid out in a Completely Randomized Design (CRD) comprising 5 levels of AHO, with three replicates per treatment. To verify the assumptions required for parametric analysis, data normality and homogeneity of variances were assessed using the D’Agostino–Pearson and Bartlett’s tests, respectively. The data obtained from the morphological and biochemical evaluations were subjected to a one-way analysis of variance (ANOVA) utilizing CoStat software (Version 6.4). subsequent mean comparisons were performed using Duncan’s multiple range test at a significance level of *p* ≤ 0.05 [79]. The charts were designed, and the standard error of the mean (±SEM) was determined via Microsoft Excel Professional Plus (2013). The statistical analyses were carried out with CoStat software (V6.4). The structural analysis and visualization of the protein–ligand complexes were performed using a combination of computational tools. The PyMOL Molecular Graphics System, Version 3.0, Schrödinger, LLC. This method was utilized for 3D molecular visualization and high-resolution image generation. Comprehensive mapping of the binding modes and characterization of the noncovalent interaction networks were executed via the BIOVIA Discovery Studio Visualizer (version 2024) and the protein–ligand interaction profiler (PLIP). Furthermore, detailed two-dimensional (2D) schematic diagrams illustrating specific hydrogen bonds and hydrophobic contacts were generated using LigPlot^+^ (v 2.3.1). A varimax-rotated principal component analysis (PCA) was conducted to evaluate the multivariate correlations among 22 traits of *K. senegalensis*. Data standardization and PCA modeling were executed with R software (version 4.5.1).

## 5. Conclusions

This study establishes AHO as a concentration-dependent biostimulant capable of optimizing plant growth and reprogramming specialized metabolic pathways. Foliar application at 20 µg/mL maximized biomass and photosynthetic pigments (chlorophylls a and b, carotenoids), alongside flavonoids and indoles, to drive carbon assimilation. Conversely, total phenolic content peaked at 1 µg/mL, indicating dose-dependent modulation of interconnected biosynthetic networks. UPLC–MS/MS profiling confirmed profound biochemical remodeling: the 10 µg/mL dose selectively upregulated chlorogenic acid and rutin, whereas the 5 µg/mL dose preferentially enriched quercetin, quercitrin, limonin, and catechin. Providing a structural basis for these findings, computational molecular docking and stereochemical analysis revealed that the S-enantiomer of AHO exhibits exceptionally high binding affinity within the active sites of key enzymes, namely chalcone synthase (CHS) and farnesyl pyrophosphate synthase (FPPS). This robust stereospecific binding establishes a direct mechanistic link between targeted secondary metabolism and enhanced physiological performance. Ultimately, AHO demonstrates significant potential as an agricultural biocatalyst that simultaneously drives crop yield and the biosynthesis of valuable secondary metabolites.

## Figures and Tables

**Table 1:** Effects of AHO application on the plant height, stem diameter, number of leaves, and root length of *K. senegalensis* seedlings.

Treatment	Plant Height (cm)	Stem Diameter (mm)	No. of Leaves	Root Length (cm)
CK	26.30 ± 1.40 c	7.30 ± 0.23 c	16.00 ± 1.53 a	23.67 ± 1.79 c
AHO 1 µg/mL	27.00 ± 1.29 c	8.51 ± 0.33 b	17.33 ± 1.45 a	24.80 ± 2.31 bc
AHO 5 µg/mL	30.33 ± 2.82 bc	8.86 ± 0.39 b	17.67 ± 1.67 a	28.57 ± 2.04 bc
AHO 10 µg/mL	32.67 ± 1.56 b	9.36 ± 0.42 ab	18.33 ± 1.86 a	30.33 ± 2.23 b
AHO 20 µg/mL	38.50 ± 1.67 a	10.11 ± 0.40 a	21.00 ± 1.53 a	39.50 ± 2.15 a
*p*-value	0.0013	0.0031	0.3301	0.0026

Note: Data are presented as the mean ± SEM (n = 3). Different letters within the same column indicate statistically significant differences between treatments according to Duncan’s multiple range test (*p* ≤ 0.05).

**Table 2:** Effects of AHO application on the fresh and dry masses of the leaves, stems and roots of *K. senegalensis* seedlings.

Treatment	Fresh Mass (g)			Dry Mass (g)		
	Leaves	Stem	Root	Leaves	Stem	Root
CK	6.43 ± 0.42 b	6.60 ± 0.50 b	6.73 ± 0.38 c	3.17 ± 0.20 b	2.57 ± 0.21 b	3.07 ± 0.23 b
AHO 1 µg/mL	9.40 ± 0.57 a	7.76 ± 0.46 ab	7.92 ± 0.38 bc	4.77 ± 0.14 a	3.20 ± 0.24 ab	3.51 ± 0.19 b
AHO 5 µg/mL	9.57 ± 0.51 a	8.03 ± 035 ab	7.97 ± 0.52 bc	4.96 ± 0.25 a	3.27 ± 0.20 ab	3.77 ± 0.22 b
AHO 10 µg/mL	9.87 ± 0.39 a	8.20 ± 0.53 a	8.47 ± 0.43 b	5.03 ± 0.30 a	3.56 ± 0.23 a	3.80 ± 0.29 b
AHO 20 µg/mL	10.93 ± 0.95 a	8.86 ± 0.47 a	10.67 ± 0.62 a	5.48 ± 0.24 a	3.80 ± 0.31 a	5.10 ± 0.23 a
*p*-value	0.0009	0.0653	0.0022	0.0003	0.0406	0.0014

Note: Data are presented as the mean ± SEM (n = 3). Different letters within the same column indicate statistically significant differences between treatments according to Duncan’s multiple range test (*p* ≤ 0.05).

**Table 4:** In silico binding affinities (kcal/mol), predicted inhibition constants (Ki), and hydrogen bonding networks of the AHO-key protein receptors.

Target Enzyme	PDB	Type	Binding Energy (kcal/mol)	Inhibition Constant (ki) µM	No. of H Bond	An Amino Acid Involved in Interaction
Chalcone Synthase (CHS)	1CGK	S	−6.43	19.26	2	Hydrogen bonds: Lys49(A), Cys84(A) Hydrophobic: Thr44(A), Asn45(A), Tyr86(A), Pro201(A)
		R	−5.65	71.92	2	Hydrogen bonds: Asn29(A), Cys30(A) Hydrophobic: Val31(A), Tyr36(A), Phe39(A), Glu74(A)
	1CGZ	S	−5.90	47.13	2	Hydrogen bonds: Gln161(A), Gly256(A) Hydrophobic: Asp255(A), Phe265(A), Pro375(A)
		R	−5.21	28.14	2	Hydrogen bonds: Thr197(A), Gly216(A) Hydrophobic: Thr132(A), Ser133(A), Cys164(A), Val193(A), Thr194(A), Phe215(A), Ser338(A)
Catechol Oxidase	1BUG	S	−6.35	22.15	1	Met 258 (A) hydrogen bond Hydrophobic: His88(A), His240(A), Ile241(A), His244(A), Asn260(A), Gly259(A), Phe261(A), Ala264(A)
		R	−5.89	48.46	1	Hydrogen bonds: Ile241(A) Hydrophobic: His88(A), His240(A), His244(A), Gly259(A), Phe261(A), Ala264(A)
Hydroxycinnamoyl-CoA Transferase (HCT)	4G0B	S	−6.47	18.13	4	Hydrogen bonds: Thr307(A), Ile309(A), Lys326(A), Arg333(A) Hydrophobic/Other: Ile201(A), Ala330(A)
		R	−5.82	54.59	1	Hydrogen bonds: Leu355(A) Hydrophobic/Other: Tyr203, Phe357
Farnesyl Diphosphate Synthase (FPPS)	1FPS	S	−7.1	6.25	2	Hydrogen bonds: Arg126(A), Gln129(A) Hydrophobic: Tyr65(A), Asn66(A), Val115(A), Cys131(A), Trp132(A)
		R	−6.26	25.62	1	Hydrogen bonds: Gln129(A) Hydrophobic: Tyr65(A), Asn66(A), Leu114(A), Val115(A), Arg126(A), Arg127(A)

**Table 3:** Identified compounds characterized by chemical formula, retention time (RT) and molecular ion (*m*/*z*) peaks using UPLC-MS/MS.

Compounds	Class	Chemical Formula	RT (min)	Mol. Ion *m*/*z*	Ion Mode
Quercetin	Flavonol	C_15_H_10_O_7_	2.48	301	[M − H]^−^
Rutin	Flavonoid Glycoside	C_27_H_30_O_16_	2.13	609.5	[M − H]^−^
Limonin	Limonoid	C_26_H_30_O_8_	2.79	469.2	[M − H]^−^
Quercitrin	Flavonoid Glycoside	C_21_H_20_O_11_	2.22	447.1	[M − H]^−^
Chlorogenic acid	Phenolic Acid	C_16_H_18_O_9_	2.01	353.1	[M − H]^−^
Catechin	Flavan-3-ol	C_15_H_14_O_6_	3.41	291.1	[M + H]^+^

**Table 5:** Synonyms, molecular formulas, smiles, 3D structures of AHO enantiomers (R and S).

Enantiomer	Molecular Formula	SMILES	Three-Dimensional Structure
*(R*)-3-acetonyl-3-hydroxyoxindole	C_11_H_11_NO_3_	CC(=O)C[C@]1(C2=CC=CC=C2NC1=O)O	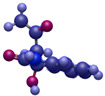
(*S*)-3-acetonyl-3-hydroxyoxindole	C_11_H_11_NO_3_	CC(=O)C[C@@]1(C2=CC=CC=C2NC1=O)O	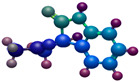

**Table 6:** Target protein profiles, biological functions, and associated pathways for molecular docking, including their corresponding PDB IDs and associated metabolic pathways.

Target Protein	PDB ID	Biological Function	Ref.
Chalcone synthase (CHS)	1CGZ	Catalyzes condensation reactions forming chalcone backbone	[59]
	1CGK	Catalyzes polyketide formation leading to flavonoids	[59]
Catechol Oxidase	1BUG	Catalyzes the key committed step in flavonoid biosynthesis via polyketide condensation reactions	[64]
Hydroxycinnamoyl-CoA Transferase (HCT)	4G0B	Catalyzes acyl transfer between hydroxycinnamoyl-CoA and quinate/shikimate	[77]
Farnesyl Diphosphate Synthase (FPPS)	1FPS	Catalyzes the synthesis of farnesyl pyrophosphate from isopentenyl pyrophosphate and dimethylallyl pyrophosphate	[78]

## Data Availability

The original contributions presented in this study are included in the article/Supplementary Materials. Further inquiries can be directed to the corresponding author.

## Supplementary Materials

The following supporting information can be downloaded at: https://www.mdpi.com/article/10.3390/ijms27157010/s1.
