# Supplementary material for: Biostimulatory Effect of 3-Acetonyl-3-Hydroxyoxindole on the Growth and Secondary Metabolites of Khaya senegalensis: UPLC–MS/MS Profiling and Molecular Docking Insights
Source: Int J Mol Sci. 2026 Aug 4;27(15):7010. doi: 10.3390/ijms27157010 (PMC13467114; doi:10.3390/ijms27157010)
Supplement: Supplementary file 1 [file ijms-27-07010-s001.zip › ijms-4433829-supplementary.pdf]

International Journal of
Molecular Sciences

MDPI

Supplementary Materials

Biostimulatory Effect of 3-Acetonyl-3-hydroxyoxindole on the Growth and Secondary Metabolites of *Khaya senegalensis*: UPLC–MS/MS Profiling and Molecular Docking Insights

Amr S. Mohamed [1,2], Yongdui Chen [2,3,4,*] and Samah M. El-Sayed [5]

1 Botanical Gardens Research Department, Horticulture Research Institute of Agricultural Research Center (ARC), Giza 12619, Egypt; amr1982@arc.sci.eg or amr_amz_9@yahoo.com
2 Biotechnology and Germplasm Resources Institute, Yunnan Academy of Agricultural Sciences, Kunming 650205, China
3 Yunnan Provincial Key Laboratory of Agricultural Biotechnology, Kunming 650205, China
4 Key Lab of South Crop Gene Resources and Germplasm Innovation, Ministry of Agricultural and Rural Affairs, Kunming 650205, China
5 Ornamental Plants and Woody Trees Department, Agricultural and Biological Research Institute, National Research Centre, Giza 12622, Egypt; ensamah_83@hotmail.com
* Correspondence: yongduichen@126.com

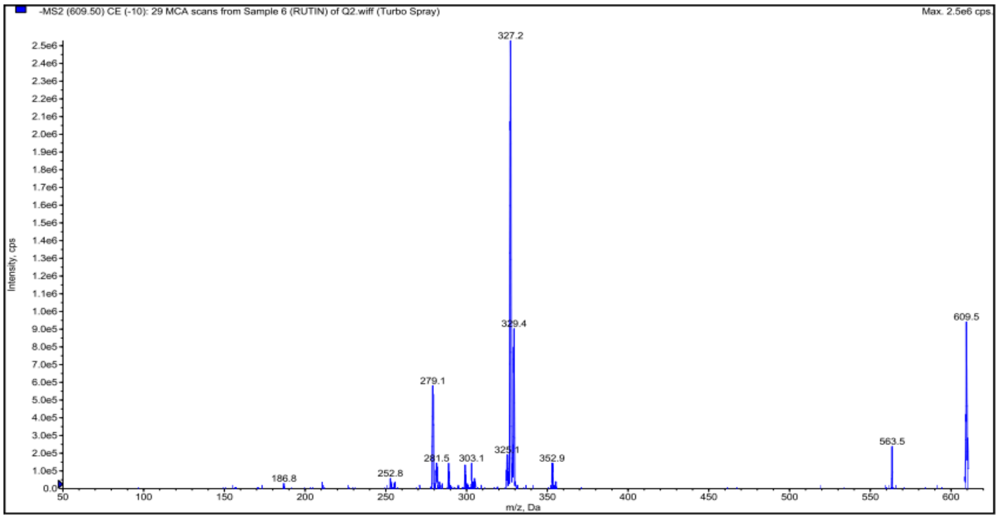

(A)

 https://doi.org/10.3390/ijms27157010

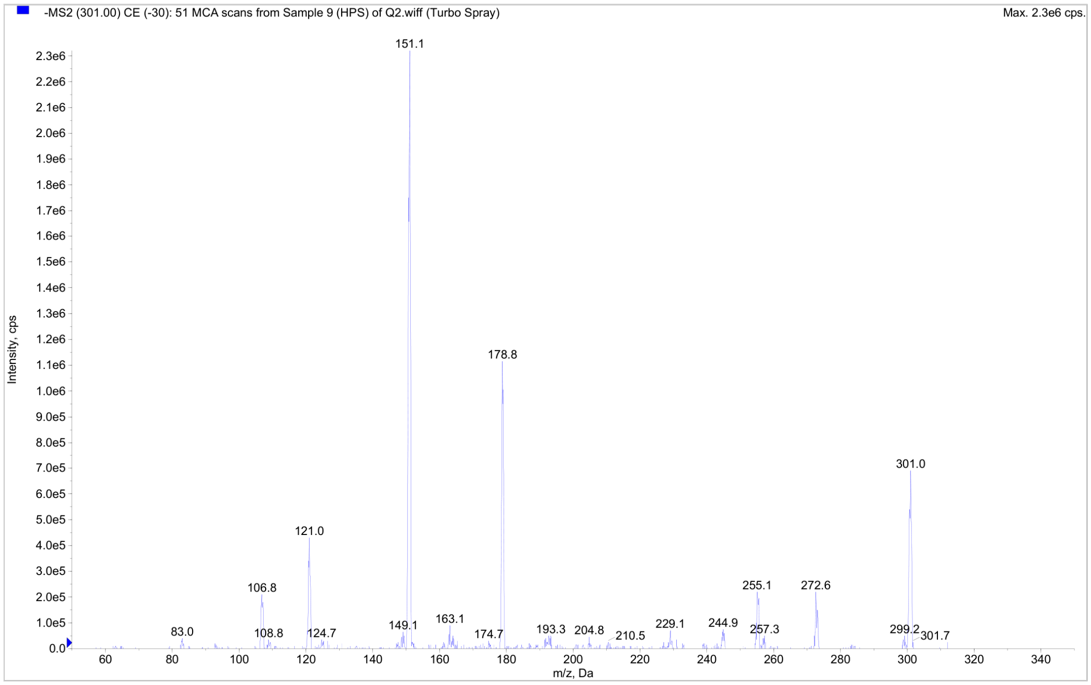
-MS2 (301.00) CE (-30): 51 MCA scans from Sample 9 (HPS) of Q2.wiff (Turbo Spray)
Max. 2.3e6 cps.
Intensity, cps
m/z, Da
151.1
178.8
301.0
121.0
106.8
255.1
272.6
83.0
108.8
124.7
149.1
163.1
174.7
193.3
204.8
210.5
229.1
244.9
257.3
299.2
301.7

(B)

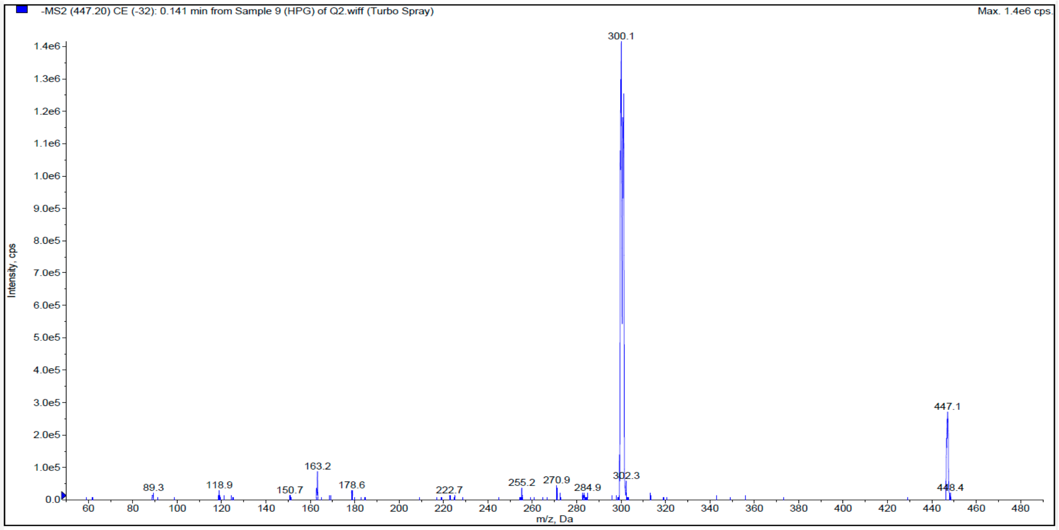
-MS2 (447.20) CE (-32): 0.141 min from Sample 9 (HPG) of Q2.wiff (Turbo Spray)
Max. 1.4e6 cps.
Intensity, cps
m/z, Da
300.1
447.1
89.3
118.9
150.7
163.2
178.6
222.7
255.2
270.9
284.9
302.3
448.4

(C)

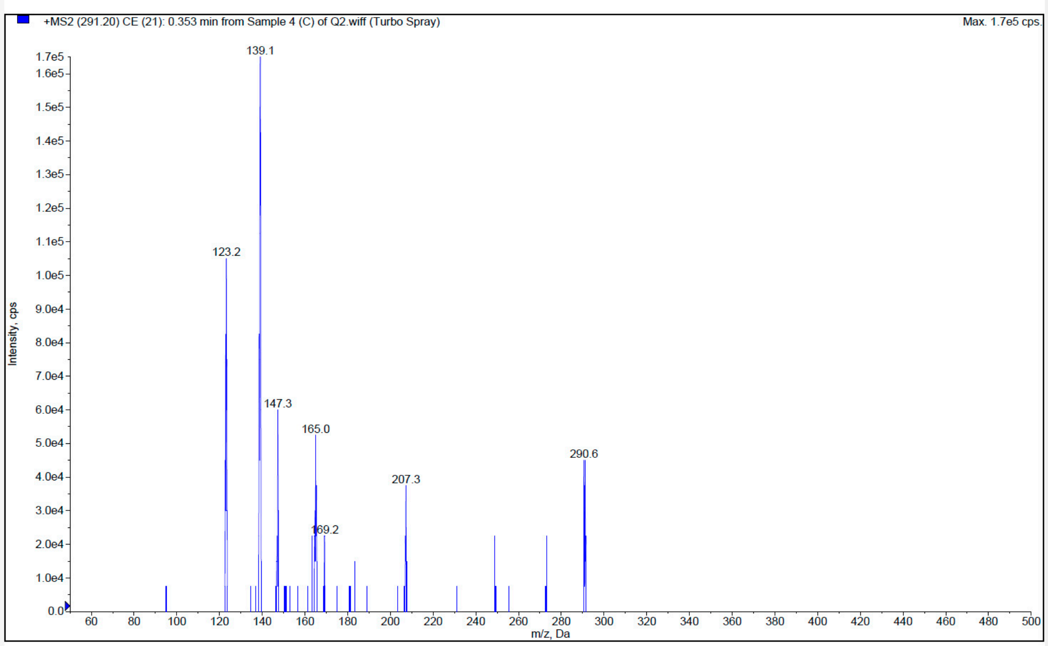
+MS2 (291.20) CE (21): 0.353 min from Sample 4 (C) of Q2.wiff (Turbo Spray)
Max. 1.7e5 cps.
Intensity, cps
m/z, Da
139.1
123.2
147.3
165.0
169.2
207.3
290.6

(D)

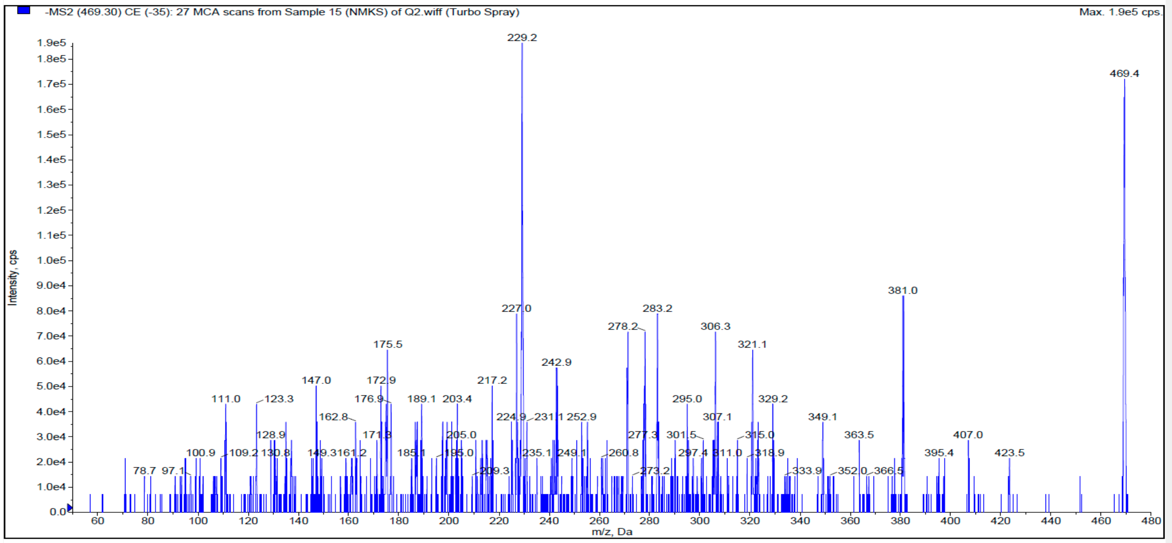
-MS2 (469.30) CE (-35): 27 MCA scans from Sample 15 (NMKS) of Q2.wiff (Turbo Spray)
Max. 1.9e5 cps.
Intensity, cps
m/z, Da
229.2
469.4
381.0
227.0
283.2
278.2
306.3
321.1
175.5
242.9
147.0
172.9
217.2
111.0
123.3
176.9
189.1
203.4
295.0
329.2
349.1
363.5
407.0
395.4
423.5

(E)

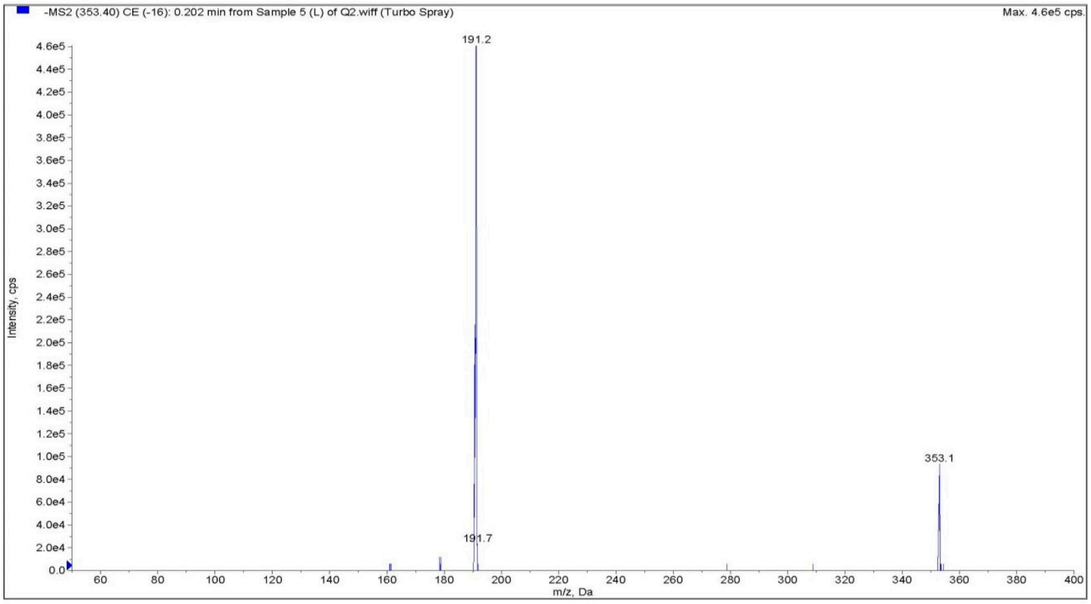

(F)

Figure S1. Characteristic ESI-MS/MS product ion mass spectra of the isolated compounds under positive and negative ionization conditions, (A): Rutin; (B): Quercetin; (C): Quercitrin; (D): Catechin; (E): Limonin; and (F): Cholorogenic acid

	Sample Name	Sample ID	Sample Type	File Name	Analyte Peak Area (counts)	Analyte Peak Height (cps)	Analyte Concentration (ng/mL)	Standard Query Status	Use Record	Record Modified
1	Sample002		Blank	Data5R112414-Hu	1.65e+001	1.42e+001	0.00	N/A		☐
2	SD0.1		Standard	Data5R112414-Hu	3.47e+002	5.36e+001	1.00	N/A	☐	☐
3	SD0.2		Standard	Data5R112414-Hu	5.16e+002	8.15e+001	2.00	N/A	☐	☐
4	SD0.5		Standard	Data5R112414-Hu	1.37e+003	1.80e+002	5.00	N/A	☐	☐
5	SD1		Standard	Data5R112414-Hu	3.80e+003	3.88e+002	10.0	N/A	☐	☐
6	SD2		Standard	Data5R112414-Hu	5.53e+003	6.29e+002	20.0	N/A	☑	☐
7	SD5		Standard	Data5R112414-Hu	1.35e+004	1.53e+003	50.0	N/A	☑	☐
8	SD10		Standard	Data5R112414-Hu	2.86e+004	3.17e+003	100.	N/A	☑	☐
9	SD20		Standard	Data5R112414-Hu	5.80e+004	6.19e+003	200.	N/A	☐	☐
10	SD50		Standard	Data5R112414-Hu	1.40e+005	1.47e+004	500.	N/A	☑	☐
11	SD100		Standard	Data5R112414-Hu	2.88e+005	2.95e+004	1000.	N/A	☑	☐
12	SD200		Standard	Data5R112414-Hu	5.85e+005	5.91e+004	2000.	N/A	☑	☐
13	QC1		Quality Control	Data5R112414-Hu	1.39e+004	1.64e+003	50.0	N/A	☑	☐
14	QC2		Quality Control	Data5R112414-Hu	1.42e+004	1.65e+003	50.0	N/A	☑	☐

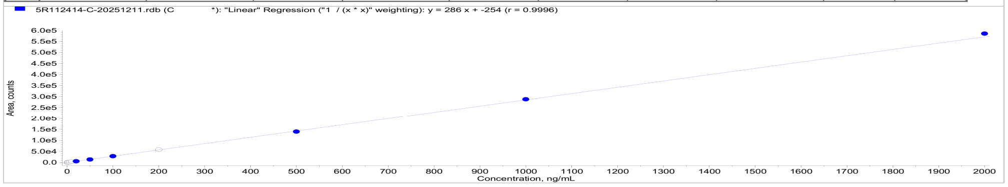

Catechin

	Sample Name	Sample ID	Sample Type	File Name	Analyte Peak Area (counts)	Analyte Peak Height (cps)	Analyte Concentration (ng/mL)	Standard Query Status	Use Record	Record Modified
1	Sample002		Blank	Data5R112414-Hu	2.19e+002	3.75e+001	0.00	N/A		☐
2	SD0.1		Standard	Data5R112414-Hu	3.53e+002	8.19e+001	0.100	N/A	☐	☐
3	SD0.2		Standard	Data5R112414-Hu	5.57e+002	1.07e+002	0.200	N/A	☐	☐
4	SD0.5		Standard	Data5R112414-Hu	9.44e+002	1.80e+002	0.500	N/A	☐	☐
5	SD1		Standard	Data5R112414-Hu	1.59e+003	3.26e+002	1.00	N/A	☐	☐
6	SD2		Standard	Data5R112414-Hu	3.20e+003	6.70e+002	2.00	N/A	☐	☐
7	SD5		Standard	Data5R112414-Hu	7.00e+003	1.52e+003	5.00	N/A	☑	☐
8	SD10		Standard	Data5R112414-Hu	1.52e+004	3.19e+003	10.0	N/A	☑	☐
9	SD20		Standard	Data5R112414-Hu	2.94e+004	6.32e+003	20.0	N/A	☑	☐
10	SD50		Standard	Data5R112414-Hu	7.29e+004	1.54e+004	50.0	N/A	☑	☐
11	SD100		Standard	Data5R112414-Hu	1.48e+005	3.17e+004	100.	N/A	☑	☐
12	SD200		Standard	Data5R112414-Hu	2.85e+005	6.18e+004	200.	N/A	☑	☐
13	QC1		Quality Control	Data5R112414-Hu	7.51e+003	1.59e+003	5.00	N/A	☑	☐
14	QC2		Quality Control	Data5R112414-Hu	6.82e+003	1.46e+003	5.00	N/A	☑	☐

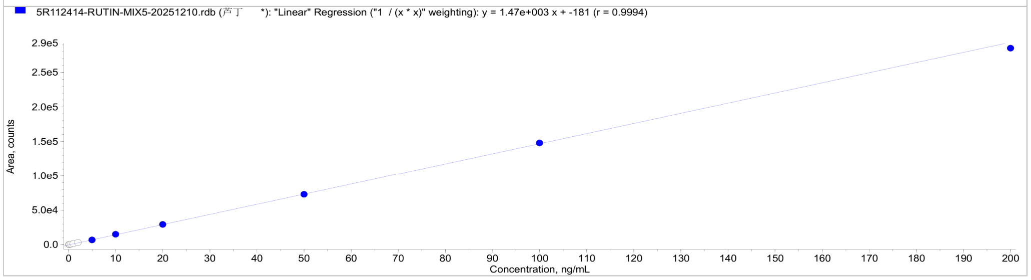

Rutin

	Sample Name	Sample ID	Sample Type	File Name	Analyte Peak Area (counts)	Analyte Peak Height (cps)	Analyte Concentration (ng/mL)	Standard Query Status	Use Record	Record Modified
1	Sample002		Blank	Data5R112414-Hu	1.43e+003	2.30e+002	0.00	N/A		☐
2	SD0.1		Standard	Data5R112414-Hu	2.26e+003	2.88e+002	0.100	N/A	☐	☐
3	SD0.2		Standard	Data5R112414-Hu	3.49e+003	3.33e+002	0.200	N/A	☐	☐
4	SD0.5		Standard	Data5R112414-Hu	2.48e+003	3.91e+002	0.500	N/A	☐	☐
5	SD1		Standard	Data5R112414-Hu	3.58e+003	6.88e+002	1.00	N/A	☐	☐
6	SD2		Standard	Data5R112414-Hu	5.79e+003	1.06e+003	2.00	N/A	☑	☐
7	SD5		Standard	Data5R112414-Hu	1.41e+004	2.56e+003	5.00	N/A	☑	☐
8	SD10		Standard	Data5R112414-Hu	2.73e+004	4.91e+003	10.0	N/A	☑	☐
9	SD20		Standard	Data5R112414-Hu	5.11e+004	9.53e+003	20.0	N/A	☑	☐
10	SD50		Standard	Data5R112414-Hu	1.22e+005	2.25e+004	50.0	N/A	☑	☐
11	SD100		Standard	Data5R112414-Hu	2.30e+005	4.16e+004	100.	N/A	☑	☐
12	SD200		Standard	Data5R112414-Hu	4.38e+005	8.01e+004	200.	N/A	☐	☐
13	QC1		Quality Control	Data5R112414-Hu	1.34e+004	2.46e+003	5.00	N/A	☑	☑
14	QC2		Quality Control	Data5R112414-Hu	1.30e+004	2.46e+003	5.00	N/A	☑	☐

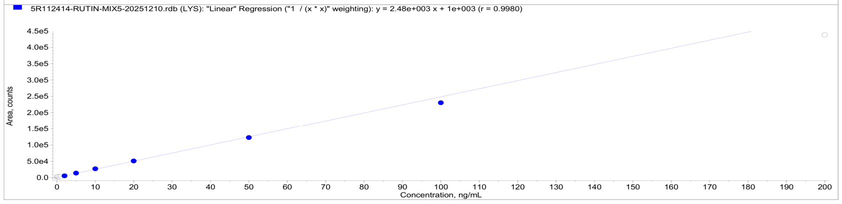

Cholorgenic acid

	Sample Name	Sample ID	Sample Type	File Name	Analyte Peak Area (counts)	Analyte Peak Height (cps)	Analyte Concentration (ng/mL)	Standard Query Status	Use Record	Record Modified
1	Sample002		Blank	Data5R112414-Hu	2.38e+001	8.33e+000	0.00	N/A		☐
2	SD0.1		Standard	Data5R112414-Hu	2.31e+002	6.31e+001	1.00	N/A	☐	☐
3	SD0.2		Standard	Data5R112414-Hu	2.72e+002	7.43e+001	2.00	N/A	☐	☐
4	SD0.5		Standard	Data5R112414-Hu	9.36e+002	2.13e+002	5.00	N/A	☑	☐
5	SD1		Standard	Data5R112414-Hu	1.48e+003	4.14e+002	10.0	N/A	☐	☐
6	SD2		Standard	Data5R112414-Hu	3.43e+003	7.74e+002	20.0	N/A	☑	☐
7	SD5		Standard	Data5R112414-Hu	8.29e+003	1.96e+003	50.0	N/A	☑	☐
8	SD10		Standard	Data5R112414-Hu	1.59e+004	3.82e+003	100.	N/A	☑	☐
9	SD20		Standard	Data5R112414-Hu	3.33e+004	7.80e+003	200.	N/A	☑	☐
10	SD50		Standard	Data5R112414-Hu	8.02e+004	1.86e+004	500.	N/A	☑	☐
11	SD100		Standard	Data5R112414-Hu	1.62e+005	3.67e+004	1000.	N/A	☐	☐
12	SD200		Standard	Data5R112414-Hu	3.00e+005	6.76e+004	2000.	N/A	☐	☐
13	QC1		Quality Control	Data5R112414-Hu	8.98e+003	2.13e+003	50.0	N/A	☑	☐
14	QC2		Quality Control	Data5R112414-Hu	8.97e+003	1.98e+003	50.0	N/A	☑	☐

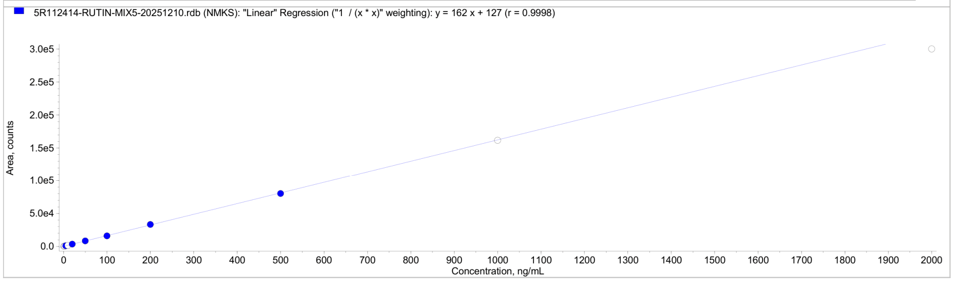

limonin

	Sample Name	Sample ID	Sample Type	File Name	Analyte Peak Area (counts)	Analyte Peak Height (cps)	Analyte Concentration (ng/mL)	Standard Query Status	Use Record	Record Modified
1	Sample002		Blank	Data5R112414-Hu	2.80e+001	1.25e+001	0.00	N/A		☑
2	SD0.1		Standard	Data5R112414-Hu	6.05e+002	1.65e+002	0.100	N/A	☑	☑
3	SD0.2		Standard	Data5R112414-Hu	8.42e+002	1.51e+002	0.200	N/A	☑	☑
4	SD0.5		Standard	Data5R112414-Hu	1.53e+003	3.33e+002	0.500	N/A	☑	☐
5	SD1		Standard	Data5R112414-Hu	3.87e+003	7.08e+002	1.50	N/A	☑	☑
6	SD2		Standard	Data5R112414-Hu	4.90e+003	8.68e+002	2.00	N/A	☑	☑
7	SD5		Standard	Data5R112414-Hu	1.11e+004	1.73e+003	5.00	N/A	☑	☐
8	SD10		Standard	Data5R112414-Hu	3.37e+004	5.80e+003	10.0	N/A	☐	☐
9	SD20		Standard	Data5R112414-Hu	6.17e+004	1.07e+004	20.0	N/A	☐	☐
10	SD50		Standard	Data5R112414-Hu	1.57e+005	2.75e+004	50.0	N/A	☐	☐
11	SD100		Standard	Data5R112414-Hu	4.08e+005	7.01e+004	100.	N/A	☐	☐
12	SD200		Standard	Data5R112414-Hu	8.44e+005	1.42e+005	200.	N/A	☐	☐
13	QC1		Quality Control	Data5R112414-Hu	1.21e+004	2.89e+003	5.00	N/A	☑	☑
14	QC2		Quality Control	Data5R112414-Hu	1.13e+004	2.15e+003	5.00	N/A	☑	☑

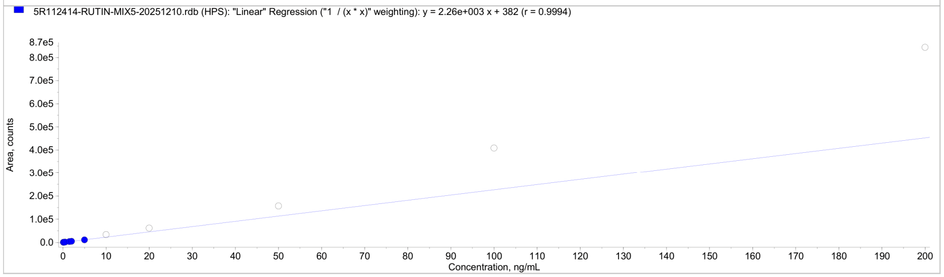

Quercetin

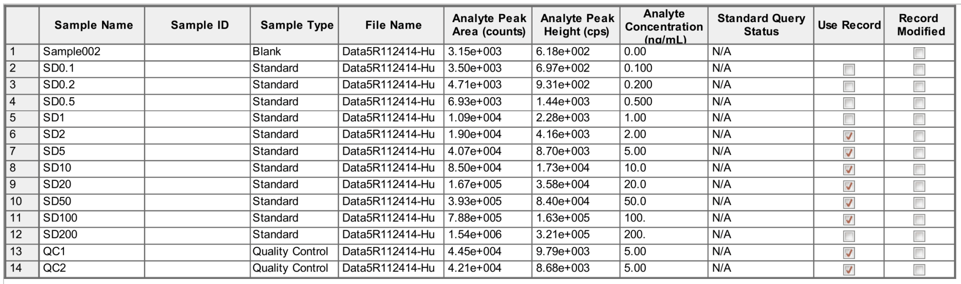

	Sample Name	Sample ID	Sample Type	File Name	Analyte Peak Area (counts)	Analyte Peak Height (cps)	Analyte Concentration (ng/mL)	Standard Query Status	Use Record	Record Modified
1	Sample002		Blank	Data5R112414-Hu	3.15e+003	6.18e+002	0.00	N/A		☐
2	SD0.1		Standard	Data5R112414-Hu	3.50e+003	6.97e+002	0.100	N/A	☐	☐
3	SD0.2		Standard	Data5R112414-Hu	4.71e+003	9.31e+002	0.200	N/A	☐	☐
4	SD0.5		Standard	Data5R112414-Hu	6.93e+003	1.44e+003	0.500	N/A	☐	☐
5	SD1		Standard	Data5R112414-Hu	1.09e+004	2.28e+003	1.00	N/A	☐	☐
6	SD2		Standard	Data5R112414-Hu	1.90e+004	4.16e+003	2.00	N/A	☑	☐
7	SD5		Standard	Data5R112414-Hu	4.07e+004	8.70e+003	5.00	N/A	☑	☐
8	SD10		Standard	Data5R112414-Hu	8.50e+004	1.73e+004	10.0	N/A	☑	☐
9	SD20		Standard	Data5R112414-Hu	1.67e+005	3.58e+004	20.0	N/A	☑	☐
10	SD50		Standard	Data5R112414-Hu	3.93e+005	8.40e+004	50.0	N/A	☑	☐
11	SD100		Standard	Data5R112414-Hu	7.88e+005	1.63e+005	100.	N/A	☑	☐
12	SD200		Standard	Data5R112414-Hu	1.54e+006	3.21e+005	200.	N/A	☐	☐
13	QC1		Quality Control	Data5R112414-Hu	4.45e+004	9.79e+003	5.00	N/A	☑	☐
14	QC2		Quality Control	Data5R112414-Hu	4.21e+004	8.68e+003	5.00	N/A	☑	☐

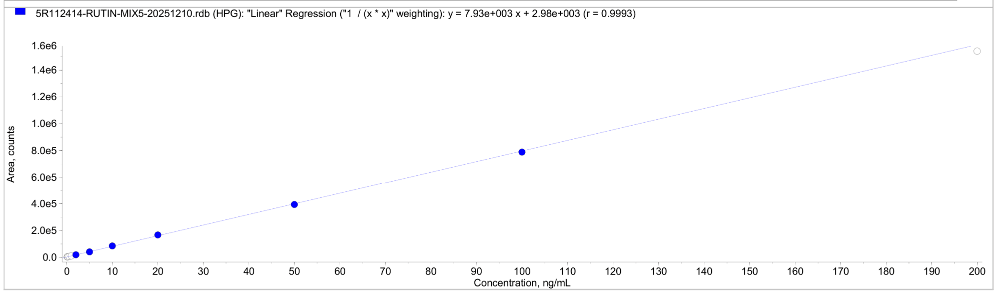

Quercitrin

Figure S2. UPLC-MS/MS calibration curve and standard calibration data for Catechin, rutin, Chlorogenic Acid, Limonin , Quercetin and Quercitrin quantification.

Table S1. Analytical method validation parameters for the targeted metabolites.

compound	Regression Equation	Correlation Coefficient (r)	Linear Range (ng/mL)	Empirical LOQ (ng/mL)	Mean Recovery (%)
**Catechin**	y = 286 x + -254	0.9996	1.00 –2000	1.00	98.80
**Chlorogenic Acid**	y = 2.48e+003 x + 1e+003	0.9980	0.100 –200	0.100	97.13
**Limonin**	y = 162 x + 127	0.9998	1.00 –2000	1.00	107.80
**Quercetin**	y = 2.26e+003 x + 382	0.9994	0.100 –200	0.100	99.00
**Quercitrin**	y = 7.93e+003 x + 2.98e+003	0.9993	0.100 –200	0.100	102.67
**Rutin**	y = 1.47e+003 x + -181	0.9994	0.100 –200	0.100	99.73

Disclaimer/Publisher's Note: The statements, opinions and data contained in all publications are solely those of the individual author(s) and contributor(s) and not of MDPI and/or the editor(s). MDPI and/or the editor(s) disclaim responsibility for any injury to people or property resulting from any ideas, methods, instructions or products referred to in the content.
